# Modeling the Effect of Microbially Induced Calcium Carbonate Precipitation (MICP) on CO_2_ Trapping

**DOI:** 10.1021/acs.est.5c08890

**Published:** 2025-10-02

**Authors:** Raymond Chen, Ahmet Mert Kavala, Alexandra Clarà Saracho, Ewa J. Marek

**Affiliations:** † Department of Chemical Engineering and Biotechnology, 2152 University of Cambridge, Cambridge CB3 0AS, U.K.; ‡ Department of Civil, Architectural and Environmental Engineering, 12330 The University of Texas at Austin, Austin, Texas 78712, United States

**Keywords:** climate change, CO_2_ sequestration, microbially induced calcium carbonate precipitation, microbial metabolism, mineral trapping, solubility trapping, pH buffers, sedimentary rock reservoirs

## Abstract

Microbially induced calcium carbonate (CaCO_3_) precipitation (MICP) is hypothesized to accelerate mineral and solubility trapping of CO_2_(g) through bacterial hydrolysis of urea, which increases pH, and hence the solubility of carbonate ions. While previous models of MICP only targeted selected conditions and did not offer modeling of all reaction kinetics, enzyme activities, and buffers in the cultivation media, our model addressed these research gaps and helped to understand the limitations and effectiveness of MICP to enhance CO_2_(g) solubility and mineral trapping. Results showed the capability of ureolysis to increase solubility trapping, with buffers in the media having a non-negligible influence on the process. However, ureolysis above pH 8.9 decreases the capacity of solubility trapping and ultimately causes CO_2_(g) outgassing. For the modeled configurations, MICP does not increase CO_2_(g) mineral trapping, since the pH increase by ureolysis is insufficient to precipitate additional CaCO_3_ than from C atoms released from urea hydrolysis. However, mineral trapping in actual sedimentary reservoirs is more complex. Thus, MICP might enhance mineral trapping in combination with mechanisms in sedimentary reservoirs, while CO_2_(g) solubility trapping by ureolysis and bacterial carbonic anhydrase enzymes can act as an important intermediate step for subsequent geochemical reactions, leading to long-term mineral trapping.

## Graphical abstract

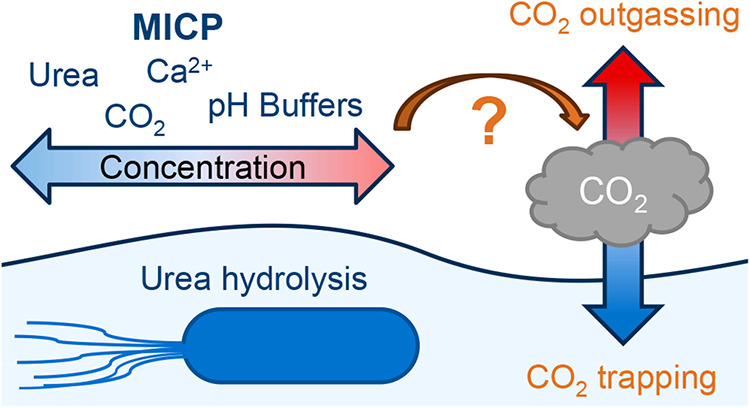

## Introduction

Reaching net-zero global CO_2_(g) emissions is essential to mitigate global warming. Yet, to prevent anticipated environmental feedback effects, net-negative CO_2_(g) emissions might be required.^1^ Carbon capture and storage (CCS) in geological formations is an available technology to remove CO_2_(g) emissions.^2^ Injection of CO_2_ into geological sites has already been implemented at scale as an enhanced oil recovery (EOR). However, unlike EOR, where long-term containment is of less concern, the permanence of CO_2_ defines the purpose of non-EOR reservoirs.^3,4^ Past CCS efforts have primarily focused on storing CO_2_ in deep saline reservoirs (e.g., >700 m) as a supercritical fluid,^5^ requiring pressures above 7.4 MPa (73 atom) and temperatures above 31 °C. CO_2_ in the supercritical state is less dense than brine,^6^ causing it to rise by buoyancy forces until it encounters a low-permeability seal, called caprock. While this method can be effective, full-scale CCS has not been widely implemented due to the lack of suitable reservoirs at the point of CO_2_ capture, concerns that buoyancy forces will allow CO_2_ to escape through caprocks, the risk of induced seismicity during CO_2_ injection, and the added costs to electricity for consumers.^7–9^ A promising but overlooked alternative is to store CO_2_ at lower pressures and temperatures (<31 °C) in shallower aquifers (e.g., <500 m), where it can be injected as a more neutrally buoyant liquid. Suitable aquifers for liquid CO_2_ storage in the US (e.g., shallow groundwater with high mineral content, unsuitable for drinking) are abundant potential CO_2_ storage sites and rarely adjacent to basement rock, where seismic events often originate.^10^ Also, well material, installation, and operation costs are considerably lower at shallower depths. However, new drilling efforts and abandoned wells are more likely in shallower aquifers, creating the risk that these storage locations will be exposed to atmospheric pressure and release gas-phase CO_2_. Hence, liquid CO_2_ must be more permanently sequestered via dissolution into water, as well as subsequent precipitation as solid minerals.^11,12^ Liquid CO_2_ dissolution into pore water at reservoir scales can take hundreds to tens of thousands of years without intervention,^13^ motivating the development of new approaches to hasten this process and mitigate risk. Converting the sequestered CO_2_ into solid carbonate species, so-called mineral trapping, or into soluble HCO_3_^–^ and CO_3_^2–^ ions in a liquid medium, so-called solubility trapping, provides a more permanent and stable form of storage.^14,15^

Microbially induced calcium carbonate (CaCO_3_) precipitation (MICP) is the process in which the urease (Ur) enzyme in ureolytic bacteria hydrolyzes urea to form ammonia and carbonic acid

R1 $$\mathrm{CO}{\left({\mathrm{NH}}_{2}\right)}_{2}+H_{2}O\overset{\mathrm{Ur}}{\to }{\mathrm{NH}}_{2}\mathrm{COOH}+{\mathrm{NH}}_{3}\left(\mathrm{aq}\right)$$

R2 $${\mathrm{NH}}_{2}\mathrm{COOH}+H_{2}O\to {\mathrm{NH}}_{3}\left(\mathrm{aq}\right)+H_{2}{\mathrm{CO}}_{3}$$

The resultant ammonia is either hydrolyzed, increasing pH (R3 in Table 1), or outgases (R4), while the carbonic acid hydrolyzes to bicarbonate (R5), and further transforms into carbonate (R6) species. In the presence of calcium ions, carbonate ions precipitate as CaCO_3_ minerals ([Disp-formula eq3]) completing the MICP process.^26–29^

R7 $${\mathrm{Ca}}^{2+}+{\mathrm{CO}}_{3}^{2-}↔{\mathrm{CaCO}}_{3}↓$$

**Table 1:** Rate and Equilibrium Constants for All Reactions Considered Relevant to MICP Modeling^a^

label	reaction	forward rate constant *k*_f_	equilibrium constant $K=\frac{k_{f}}{k_{b}}$
R1	$\mathrm{CO}{\left({\mathrm{NH}}_{2}\right)}_{2}+H_{2}O\to {\mathrm{NH}}_{2}\mathrm{COOH}+{\mathrm{NH}}_{3}\left(\mathrm{aq}\right)$	3.2 × 10^–21^ s^–1^ ^17^	^d^
R1 (catalyzed by Ur)	$\mathrm{CO}{\left({\mathrm{NH}}_{2}\right)}_{2}+H_{2}O\overset{\mathrm{Ur}}{\to }{\mathrm{NH}}_{2}\mathrm{COOH}+{\mathrm{NH}}_{3}\left(\mathrm{aq}\right)$	*cf*. eq 6	^d^
R2	${\mathrm{NH}}_{2}\mathrm{COOH}+H_{2}O\to {\mathrm{NH}}_{3}\left(\mathrm{aq}\right)+H_{2}{\mathrm{CO}}_{3}$	1 × 10^10^ s^–1^ ^c^	^d^
R3	${\mathrm{NH}}_{3}\left(\mathrm{aq}\right)+H_{2}O↔{\mathrm{NH}}_{4}^{+}+{\mathrm{OH}}^{-}$	1 × 10^10^ s^–1^ ^c^	1.8 × 10^–5^ M^18^
R4	${\mathrm{NH}}_{3}\left(\mathrm{aq}\right)↔{\mathrm{NH}}_{3}\left(g\right)$	1 × 10^10^ s^–1^ ^c^	6.8 × 10^–4^ ^19^
R5	$H_{2}{\mathrm{CO}}_{3}↔{\mathrm{HCO}}_{3}^{-}+H^{+}$	1 × 10^7^ s^–1^ ^20^	*K*_*R*5_ = 1.7 × 10^–4^ M^20^
R6	${\mathrm{HCO}}_{3}^{-}↔{\mathrm{CO}}_{3}^{2-}+H^{+}$	3 s^–1^ ^21^	*K*_2_ = 4.68 × 10^–11^ M
R7	Ca^2+^ + CO_3_^2–^ ↔ CaCO_3_	2 s^–1^ M^–1^ ^21^	*K*_*R*7_ = 2 × 10^8^ M^–1^ ^22^
R8	${\mathrm{CO}}_{2}\left(g\right)↔{\mathrm{CO}}_{2}\left(\mathrm{aq}\right)$	2 × 10^–2^ s^–1^ ^23^ ^b^	8.4 × 10^–1^ ^19^
R9	${\mathrm{CO}}_{2}\left(\mathrm{aq}\right)+H_{2}O\overset{\mathrm{CA}}{↔}H_{2}{\mathrm{CO}}_{3}$	0.065 s^–1^ + 0.117 s^–1^ × [*X*_*OD*_], *cf*. eq 7	*K*_1_/*K*_*R*5_ = 2.63 × 10^–3^
R10	$H^{+}+{\mathrm{OH}}^{-}↔H_{2}O$	1.4 × 10^11^ s^–1^ M^–1^ ^24^	$1/K_{w}=10^{14}\frac{1}{M^{2}}$
R11	${\mathrm{HCO}}_{3}^{-}+{\mathrm{OH}}^{-}↔{\mathrm{CO}}_{3}^{2-}+H_{2}O$	6 × 10^9^ s^–1^ M^–1^ ^25^	*K*_2_/*K*_*w*_ = 4.68 × 10^3^ M^–1^
R12	${\mathrm{CO}}_{2}\left(\mathrm{aq}\right)+{\mathrm{OH}}^{-}↔{\mathrm{HCO}}_{3}^{-}$	8.5 × 10^3^ s^–1^ M^–1^ ^25^	*K*_1_/*K*_*w*_ = 4.47 × 10^7^ M^–1^
R13	$\mathrm{HQa}↔{\mathrm{Qa}}^{-}+H^{+}$	1 × 10^10^ s^–1^ ^c^	10^–*pK*_*a*,Qa–_^ = 2.0 × 10^–10^ M
R14	$\mathrm{HQb}↔{\mathrm{Qb}}^{-}+H^{+}$	1 × 10^10^ s^–1^ ^c^	10^–*pK*_*a*,Qb–_^ = 5.0 × 10^–9^ M
R15	$\mathrm{HQc}↔{\mathrm{Qc}}^{-}+H^{+}$	1 × 10^10^ s^–1^ ^c^	10^–*pK*_*a*,Qc–_^ = 1.0 × 10^–6^ M
R16	$\mathrm{HQd}↔{\mathrm{Qd}}^{-}+H^{+}$	1 × 10^10^ s^–1^ ^c^	10^–*pK*_*a*,Qd–_^ = 6.3 × 10^–5^ M
R17	$\mathrm{HQe}↔{\mathrm{Qe}}^{-}+H^{+}$	1 × 10^10^ s^–1^ ^c^	10^–*pK*_*a*,Qe–_^ = 1.3 × 10^–4^ M
R18	$\mathrm{HQf}↔{\mathrm{Qf}}^{-}+H^{+}$	1 × 10^10^ s^–1^ ^c^	10^–*pK*_*a*,Qf–_^ = 4.0 × 10^–3^ M
R19	$\mathrm{HQg}↔{\mathrm{Qg}}^{-}+H^{+}$	1 × 10^10^ s^–1^ ^c^	10^–*pK*_*a*,Qg–_^ = 7.9 × 10^–8^ M

a The values are given at ∼25 °C. The equilibrium constants of the buffers are based on *pK*_*a*_ values from amino acids in the media. The first and second dissociation constants of carbonic acid $K_{1}=\frac{[{\mathrm{HCO}}_{3}^{-}]\left[H^{+}\right]}{\left[{\mathrm{CO}}_{2}\left(\mathrm{aq}\right)\right]}=4.47\times 10^{-7}M$ and $K_{2}=\frac{[{\mathrm{CO}}_{3}^{2-}]\left[H^{+}\right]}{\left[{\mathrm{HCO}}_{3}^{-}\right]}=4.68\times 10^{-11}M$ , and the dissociation constant of water *K*_w_ = [H^+^]­[OH^–^] = 10^–14^ M^2^ are taken from Zeebe and Wolf-Gladrow.^16^

b Based on an experimental value for oxygen transfer in bottles in a shaking incubator,^23^ value is only an estimate and not necessarily applicable to natural aquifers.

c Rate constant could not be found, thus assumed to be instantaneous

d Irreversible reaction, no equilibrium constant.

The MICP process happens on a time scale of hours to days.^27,30^ In comparison, natural and abiotic trapping of CO_2_(g) to carbon-containing ions or solid minerals also progresses through the creation of H_2_CO_3_ *via* the dissolution of CO_2_(g) in water (R8) and the hydrolysis of CO_2_(aq) to H_2_CO_3_ (R9), followed by the dissociation of the H_2_CO_3_ in (R5) and (R6). However, in abiotic trapping, the mechanism to yield soluble CO_3_^2–^, HCO_3_^–^, and solid CaCO_3_ is overall much slower and spans from years to millennia.^2,31,32^

Besides a much faster route to CaCO_3_ precipitation, MICP can also enhance CO_2_(g) solubility trapping in the presence of CO_2_(g), owing to the overlap with the abiotic trapping pathway, mainly through (R3), (R5), and (R6).^28^ Here, we described the dissolution of gaseous CO_2_ in water following the reactions laid out by Mitchell et al.,^28^ although we expect a similar dissolution of liquid and supercritical CO_2_ in water, with all reactions beyond R8 following analogously. Additionally, some ureolytic bacterial strains, such as *Sporosarcina pasteurii*, produce the carbonic anhydrase (CA) enzyme,^33^ which catalyzes and accelerates R9. *S. pasteurii* is a soil-dwelling ureolytic strain that produces both the Ur and CA enzymes and is commonly used in MICP research.^30,33^ *S. pasteurii* is also able to survive in extreme temperatures and pressures and is nonpathogenic, making *S. pasteurii* a good candidate for MICP-enhanced geological CO_2_(g) storage.^27,28,30^ Therefore, we chose to focus on *S. pasteurii* as the organism used for MICP in this paper.

A possible reaction pathway of MICP interacting with the abiotic CO_2_(g) trapping route through the Ur ([Disp-formula eq1]) and CA enzymes (R9) is shown in Figure 1. Alternative pathways for MICP and CO_2_(g) interactions are also possible, for example *via* reactions R10, R11, and R12 in Table 1, and could effectively result in the same outcome; those reactions, however, have been omitted from Figure 1 for clarity. As seen in Figure 1, both the abiotic and MICP reaction routes produce H_2_CO_3_, which further dissociates to HCO_3_^–^ (R5), CO_3_^2–^ (R6), and protons H^+^, resulting in a decrease in pH. At lower pH, the equilibrium of R5 and R6 shifts to the left, inhibiting further CO_2_(g) solubility trapping in HCO_3_^–^ and CO_3_^2–^, as evidenced by the Bjerrum plot for carbonate species.^16^ However, the MICP reaction pathway can counteract this effect by increasing the pH when ammonia is hydrolyzed in R3. As a result, CO_2_(g) trapping in the form of soluble HCO_3_^–^ and CO_3_^2–^ continues, with some CO_3_^2–^ being further precipitated into CaCO_3_ minerals in R11.^16,28,36,37^ The set of all of the key reactions occurring during MICP in the presence of CO_2_(g) is provided in Table 1.

**Figure 1:**
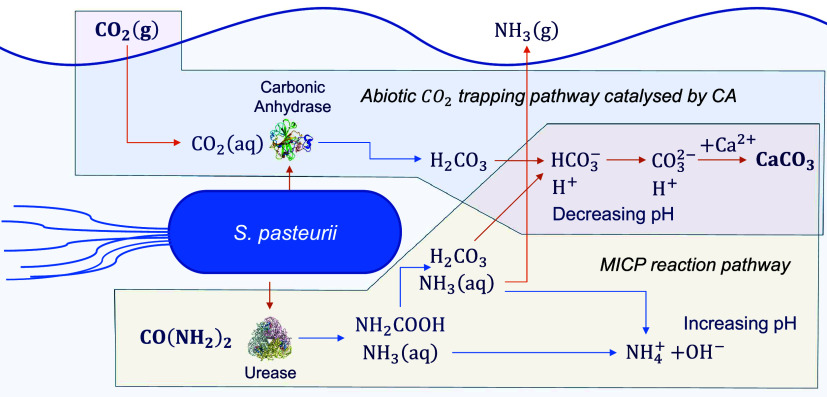
Possible reaction pathways describing the interaction of MICP with abiotic CO_2_(g) trapping where conversion of CO_2_(g) to CaCO_3_ minerals is aided by Ur and CA enzymes from *S. pasteurii*. The liquid phase is shaded with light blue, and the gas phase is in white. The shaded boxes show the scope of MICP and the abiotic trapping pathway, respectively. Reactions with blue instead of orange arrows consume one H_2_O molecule, omitted here for clarity. Not all possible reaction paths are shown; a list of all key reactions is given in Table 1. Image of CA taken from Heidarnezhad et al.^34^ and image of Ur taken from Roberts et al.^35^

The kinetics of CO_2_(g) dissolution in water and the subsequent chemical reactions leading to the formation of CaCO_3_ in an abiotic system have been previously modeled by Mitchell et al.^28^ For a biotic system with MICP, Mitchell et al.^28^ investigated the effect of MICP on carbon trapping through a combination of experimental and numerical analyses. Their model assumes ureolysis to occur in discrete, time-independent steps, followed by system equilibration after each step, thereby neglecting the reaction kinetics and enzyme activities, leaving the temporal scale of the trapping process unresolved. Including reaction kinetics and enzyme activities does not affect the final potential amount of CO_2_(g) that can be trapped, since this potential is governed by thermodynamic equilibrium rather than kinetics. Therefore, models that neglect kinetics do not necessarily misinterpret the total trapping capacity. However, incorporating reaction kinetics and enzyme activities allows for estimation of the time scales over which the trapping process occurs, enables assessment of the effects of carbonic anhydrase, and facilitates comparison and validation with experimental data. While experimental results from Mitchell et al.^28^ showed an increase of the proportion of CO_2_(g)-derived C in CaCO_3_ minerals, their model showed no net precipitation of CO_2_(g) into CaCO_3_ minerals. Nevertheless, the higher pH achieved through ureolysis led to the enhanced solubility of CO_2_(g), trapped in the form of the CO_3_^2–^ and HCO_3_^–^ ions. This nuance may have led to misinterpretations in the literature, which cite Mitchell et al.^28^ as supporting CO_2_ mineral trapping *via* MICP. Therefore, we also present a fundamental mechanistic understanding of the involved physical processes in MICP and their effect on CO_2_ mineral trapping. Furthermore, since Mitchell et al.^28^ quantified the trapping only in selected experimental conditions, the maximal extent of the possible CO_2_(g) trapping remains unclear. Further research is thus also required to systematically elucidate the amount of CaCO_3_ precipitation by MICP for varied CO_2_(g) and urea concentrations.

While previous research has focused on MICP for mineral trapping, ureolysis alone for solubility trapping has not been quantitatively assessed before for its trapping capacity and limits. In this study, we model for the first time the process of ureolysis without Ca^2+^ ions to quantify its potential for CO_2_ solubility trapping under varied conditions.

In this article, we therefore investigated the potential and limits of MICP-mediated CO_2_ trapping to understand which conditions benefit CO_2_ trapping and when MICP could lead to the adverse effects of CO_2_ outgassing. Aiming to guide further research on CO_2_ trapping with MICP, we analyzed our results to additionally provide a comprehensive understanding of the underlying mechanisms.

## Materials and Methods

### Batch Experiments to Investigate CO_2_(g) Trapping

*S. pasteurii* (DSM 33) was cultivated in closed sterilized 250 mL bottle reactors (see Figure 2), using 50 mL DSMZ 220 media^38^ supplemented with 20 g L^–1^ urea and adjusted to pH 7.3, with incubation carried out in a shaking incubator at 30 °C and 200 rpm over 54 h.

**Figure 2:**
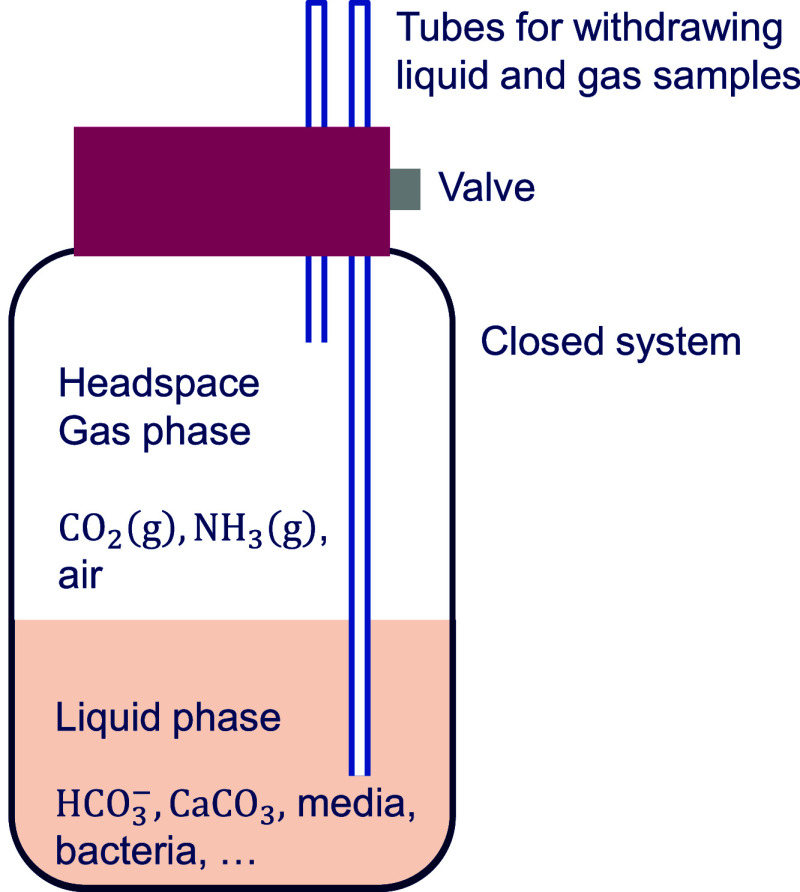
Schematic of bottle reactors, in which bacteria were cultivated for the measurement of CO_2_(g) trapping.

A urea concentration of 20 g L^–1^ (0.33 M) was chosen based on previous studies^26,39^ and on the concentration of urea-rich, industrial wastewater (e.g., from urea production), which is regarded as a cheap and abundant source of urea.^28,40,41^ Bacteria were inoculated at an initial optical density at 600 nm (OD_600_) of 0.01, measured using a UV–visible spectrophotometer (Evolution 201, Thermo Scientific). MICP takes place stoichiometrically at an equimolar concentration of urea and Ca^2+^. However, consistent with previous work,^42^ we supplied urea in excess to ensure high ureolytic efficiency driving carbonate formation, thus choosing a lower molar Ca^2+^ concentration of 0.18 M by supplying 20 g L^–1^ CaCl_2_.

The main experimental series are designated as “bacteria” when no further additions were used in the liquid phase, “bacteria + Ca” when 20 g L^–1^ CaCl_2_ was included in the media, and “control” when neither CaCl_2_ nor bacteria were used. These labels refer only to the condition of the liquid phase.

The remaining 200 mL headspace of the reactors was filled with a gas mixture of blended air (21 vol % O_2_, 79 vol % N_2_, BOC) and CO_2_(g) at atmospheric pressure, with CO_2_(g) concentrations of either 0.04, 50, or 60 vol %. Because of the existence of more accurate thermodynamic data for atmospheric pressure and the existence of experimental data for validation, we decided to examine the MICP process at atmospheric pressure (1013.25 hPa), although geological CO_2_ storage sites can have pressures above 73 atom and temperatures above 31 °C.^28,32^ The selected CO_2_ concentrations (0.04, 50, and 60%) were determined by the constraints of our experimental setup, and the limitations to measure CO_2_ accurately. However, these concentrations allowed us to test system behavior under high CO_2_ loading, which is relevant for process evaluation. The oxygen in the headspace can influence the activity of *S. pasteurii*, with an anaerobic environment upon employment of *S. pasteurii* in geological sites potentially impacting the bacterial growth and activity. However, bacteria can get acclimated to new environments^43^ and anaerobic ureolytic strains from the target environment could be isolated for employment in the geological sites.

A total of nine experimental series were conducted, encompassing all variations in the gas- and liquid-phase conditions. Thus, the experimental labels further include information about the CO_2_(g) concentrations, e.g., “bacteria 50 vol % CO_2_(g)” or “control 0.04 vol % CO_2_(g)”, with the numbers indicating the starting volumetric CO_2_(g) fraction in the headspace.

The reactors included a Diba Omnifit cap with two access ports fitted with PTFE tubes and on/off valves. One long PTFE tube extended into the liquid phase for liquid sampling, while a shorter tube extended only to the headspace to sample the gas (Figure 2). Gas samples were withdrawn using a 5 mL syringe and injected into a gas chromatograph (Agilent 7890A) to determine the CO_2_(g) concentration. Liquid samples (2 mL) withdrawn from the reactors were used to measure OD_600_ - the optical density at 600 nm. More details on the batch experiments can be found in the study of Clarà Saracho and Marek.^44^

### Modeling of Substance Concentrations during MICP

A model for tracking all species participating in the reactions listed in Table 1 was formulated and implemented in Python. For example, for a reaction A + B $\overset{k_{f}}{\underset{k_{b}}{↔}}$ C, the rate was described as

1 $$\frac{d}{\mathrm{dt}}\left[A\right]=\frac{d}{\mathrm{dt}}\left[B\right]=-\frac{d}{\mathrm{dt}}\left[C\right]=-k_{f}\left[A\right]\left[B\right]+k_{b}\left[C\right]$$

with [A], [B], and [C] being the molar concentrations of substances A, B, and C, respectively; *k*_f_ and *k*_b_ the forward and backward reaction rate constants, respectively; and

2 $$K=\frac{k_{f}}{k_{b}}=\frac{\left[C\right]}{\left[A\right]\left[B\right]}$$

the equilibrium constant. The rate equations were implemented as a coupled differential equation system and solved as an initial value problem (ivp).

The created model was used to simulate MICP and the fate of CO_2_(g) during the batch experiments in the bottles. The bottle reactors were modeled as a closed system with a liquid phase, and a headspace gas phase containing CO_2_(g), NH_3_(g), and air, modeled as ideal gases. Perfect mixing with a uniform distribution of components across each phase was assumed, because the bottles were placed in a shaking incubator. All reactions were assumed to take place in the liquid phase, with no interaction of components in the gas phase. Additionally, the mass transfer processes between the gas and liquid phases, CO_2_(g) ↔ CO_2_(aq) and NH_3_(aq) ↔ NH_3_(g) were modeled at the gas–liquid interface. Notably, the developed model is not constrained to bottles, as it is not restricted by a set volume. This allows a straightforward application to other systems provided that the stated assumptions remain true.

#### Reactions and Kinetics

All relevant reactions for modeling MICP are listed in Table 1, together with the respective equilibrium and rate constants reported for atmospheric pressure and temperatures ranging from 20 to 30 °C. The model ignored any possible heat effects from reactions and bacteria.

The dissolution of NH_3_(g) (R4) and CO_2_(g) (R8) in water was described using Henry’s law for gas solubility and Fick’s first law to calculate the flux of components between well-mixed gas and liquid phases. The resulting rate equation describing mass transfer was equivalent in form to that for a chemical reaction but applying an effective rate constant. Hence, eq 4 also presents the rate of mass transfer processes included in the model for components exchange in a quickly equilibrating, shaking incubator.^25^

Amino acids in bacterial growth media provide important buffering capacity.^45^ Since CO_2_(g) trapping is pH-dependent, we accounted for the buffering capacity of the amino acids present in the peptone from casein and soymeal in DSMZ 220 media, determining their parameters empirically, with the method detailed in the SI, section S1.1.2.^46,47^

At the start of the simulation, CO_2_(g), urea, Ca^2+^, H^+^, OH^–^, buffers, water, and air were the only molecules present. Initial concentrations for CO_2_(g), urea, and Ca^2+^ were varied depending on the configuration of the simulation. Buffers, H^+^, and OH^–^ were initialized as equilibrated with the initial media pH of 7.3.

In simulations with continuous CO_2_(g) or Ca^2+^ supply, such as modeling the continuous injection of CO_2_(g) into geological CCS storage sites, the concentration of CO_2_(g) or Ca^2+^, respectively, was kept constant.

#### Reaction Rates of Catalyzed Reactions

The rate of the irreversible, Ur-catalyzed ureolysis [Disp-formula eq1] was modeled using a growth-dependent variation of the Michaelis–Menten kinetics:

3 $$\frac{d}{\mathrm{dt}}\left[\mathrm{CO}{\left({\mathrm{NH}}_{2}\right)}_{2}\right]=-\left[X\right]\times \frac{v_{\max}\left[\mathrm{CO}{\left({\mathrm{NH}}_{2}\right)}_{2}\right]}{K_{m}+\left[\mathrm{CO}{\left({\mathrm{NH}}_{2}\right)}_{2}\right]}$$

where [X] represents the bacteria concentration in the liquid phase, expressed using colony-forming units (CFU) per mL, *K*_m_ the Michaelis constant, and *v*_max_ × [X] the maximum rate constant. For *S. pasteurii*, Lauchnor et al.^26^ obtained the values *K*_m_ = 355 mM, *v*_max_ = 6.4 × 10^–9^ mmol h^–1^ CFU^–1^. The above approach is correct only if the cell concentration, CFU mL^–1^, is proportional to the enzyme concentrations. Lauchnor et al.^26^ deemed such an assumption to be valid for the case of *S. pasteurii* because its urease enzyme is constitutively produced. They assumed a linear relationship between OD_600_ and CFU, with an OD_600_ of 1 corresponding to 1.37 × 1 × 10^9^ CFU mL^–1^. However, this linear relationship was only considered valid during the exponential phase.

With the relationship of an OD_600_ of 1 corresponding to 1.37 × 10^9^ CFU mL^–1^ from Lauchnor et al.,^26^ the amount of bacteria at a given time (i.e., [X] in CFU mL^–1^) was obtained by converting the OD_600_ measured at 0, 2, 5, 8, 24, 31, 48, 54 h during the batch experiments or by linear interpolation between these points.

Following the peak of exponential phase of bacterial growth (at 24 h for “bacteria 0.04 vol % CO_2_(g)” and “bacteria 50 vol % CO_2_(g)”, 31 h for “bacteria 60 vol % CO_2_(g)” and “bacteria + Ca 0.04 vol % CO_2_(g)”, 54 h for “bacteria + Ca 50 vol % CO_2_(g)” and “bacteria + Ca 60 vol % CO_2_(g)”), the values of [X] were set to remain constant, thus reflecting the stationary phase of bacterial life, during which experimental OD_600_ measurements were not considered linear to bacterial concentration [X] anymore. Moreover, with most of the urea present in the liquid phase being hydrolyzed by the time of the peak, slight inaccuracies in bacterial concentration [X] postpeak do not significantly impact the ureolysis rate ([Disp-formula eq1]). For the “control” experiment, [X] was set to zero. The values of [X] obtained from the experimental work and then used for modeling the batch experiments in bottles are shown in Figure 3.

**Figure 3:**
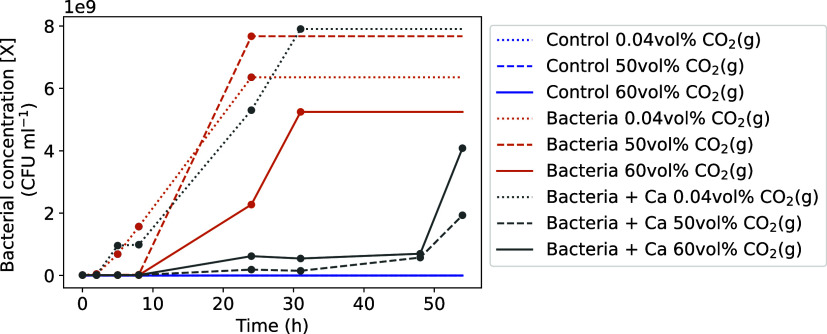
Values of bacterial concentration [X] used for modeling the batch experiments in bottles. Points are values based on actual measured OD_600_ values, and lines show the linear interpolations.

The first-order rate constant of the CA-catalyzed R9 was determined by measuring the pH change induced by the hydrolysis of CO_2_(aq), with the methodology detailed in the SI, section S1.1.3, and by Chen et al.^48^ The correlation between OD_600_ and the first-order rate constant of R9 was determined as

4 $$k_{f,\mathrm{CA}}=0.065\;s^{-1}+0.117\;s^{-1}\times \left[X_{\mathrm{OD}}\right]$$

with [*X*_OD_] being the cell concentration expressed in OD_600_. The intercept of 0.065 s^–1^ accounts for the rate of the uncatalysed R9, aligning closely with reported literature values of 0.03–0.062 s^–1^.^21,25,49^

In this study, the carbon in bacterial biomass and growth medium was assumed not to interact with other species, as the focus was on the effects of ureolysis on solubility and mineral trapping rather than CO_2_(g) trapping through bacterial growth. However, experimental measurements showed cell concentrations of up to ∼8 × 10^9^ CFU mL^–1^, and with a single cell of *S. pasteurii* containing 0.97 pg carbon (0.08 pmol),^28^ the total carbon in bacterial biomass reached up to ∼640 mM, which is not negligible. While a proportion of the carbon likely originated from the peptones in the media, some carbon could also be converted from urea or CO_2_(g) to bacterial biomass.^44^ Therefore, while the possibility of carbon being trapped in the form of bacterial biomass should not be neglected in general, this study focuses solely on carbon trapping through bacterial ureolysis.

#### Simulation of Results from the Literature

We validated our model by simulating a system investigated by Mitchell et al.^21^ and compare the results from our model with the results from Mitchell et al.,^21^ as detailed in the SI, section S1.1.4 and Figure S3.

## Results and Discussion

To support the following evaluation, we introduce the term “trapped CO_2_(g)” (τ_trapped CO_2_(g)_) as the molar concentration of all carbon atoms in the liquid phase, excluding carbon from urea, bacteria, and media:

5 $$\tau_{{\mathrm{trapped}\;\mathrm{CO}}_{2}\left(g\right)}=\tau_{C}-\left[{\mathrm{CO}}_{2}\left(g\right)\right]-\tau_{\mathrm{CO}{\left({\mathrm{NH}}_{2}\right)}_{2}}$$

where τ_C_ is the total amount of carbon atoms in the gas and liquid phase and τ_CO(NH_2_)_2__ is the sum of hydrolyzed and unhydrolyzed moles of urea at any given time.

Table 2 provides a summary of the boundary conditions and key variables used in all results figures, providing assistance to the readers.

**Table 2:** Overview of Boundary Conditions and Key Variables of Figures

	initial [urea] (M)	initial [Ca^2+^] (M)	initial CO_2_(g) fraction (vol %)	initial pH	constant headspace CO_2_(g)	media buffers included
Figure 3	0.33 (20 g L^–1^)	varied (line color)	varied (line style)	7.3	no	yes
Figure 4	0.33 (20 g L^–1^)	varied (line color)	varied (line style)	7.3	no	yes
Figure 5	0.33 (20 g L^–1^)	varied (*x*-axis)	varied (line color)	7.3	yes	no
Figure 6	0.33 (20 g L^–1^)	varied (*x*-axis)	varied (line color)	7.3	yes	no
Figure 7	0	0	50	7.3	yes	yes
Figure 8	0 (at *t* < 0); 0.33 (at *t* > 0)	0.66 (73.33 g L^–1^ CaCl_2_)	100	10^a^	yes (at *t* < 0); no (at *t* > 0)	yes^b^
Figure 9	varied (*x*-axis)	0	varied (line color)	7.3	yes	yes
Figure 10	varied (*x*-axis)	0	varied (line color)	7.3	yes	no
Figure 11	varied (not shown)	varied (line color)	varied (*x*-axis)	7.3	yes	varied (line style)
Figure 12	0.33 (20 g L^–1^)	varied (line color)	varied (*x*-axis)	7.3	yes	yes
Figure 14	0.33 (20 g L^–1^)	0	100	7.3	yes	yes
Figure S4	0.33 (20 g L^–1^)	varied (line color)	varied (line style)	7.3	no	yes
Figure S5	0.33 (20 g L^–1^)	varied (*x*-axis)	varied (line color)	7.3	yes	yes
Figure S6	0.33 (20 g L^–1^)	varied (*x*-axis)	varied (line color)	7.3	yes	yes
Figure S7	varied (*x*-axis)	0.18 (20 g L^–1^ CaCl_2_)	varied (line color)	7.3	yes	yes

a An initial configuration (with pH 10) was given, and the system was allowed to equilibrate. At *t* < 0, the equilibrated system is shown, thus having a different pH in the figure.

b additionally 0.66 M buffer with *pK*_*a*_ = 10.

### Observation of CO_2_(g) Trapping and Comparison of Experimental and Simulated Results

The comparison between the volumetric CO_2_(g) fraction from the batch experiments and the corresponding simulations is shown in Figure 4a. Generally, for the “control” and “bacteria” cases, the values toward the second half of the experiment and the shape of the curves are in good agreement. However, the decrease in volumetric CO_2_(g) fraction of the experiments with 50 and 60 vol % CO_2_(g) of the “bacteria” and “bacteria + Ca” series was later in the experiments than in the simulated results. A possible explanation for this discrepancy is an overestimation of the biomass concentration, calculated based on a linear relationship between OD_600_ and CFU mL^–1^ and consequently, an exaggerated rate of ureolysis ([Disp-formula eq1]). If a factor of 250 lower bacterial concentration is assumed, the simulated CO_2_(g) fractions align more closely with the experimental results, as shown in Figure 4b. Potential reasons for this lower bacterial concentration than the one obtained when using the OD_600_ to CFU mL^–1^ conversion from Lauchnor et al.^26^ could be the differences between the used spectrophotometers and media type, as well as culture stress and presence of secreted compounds.^50^

**Figure 4:**
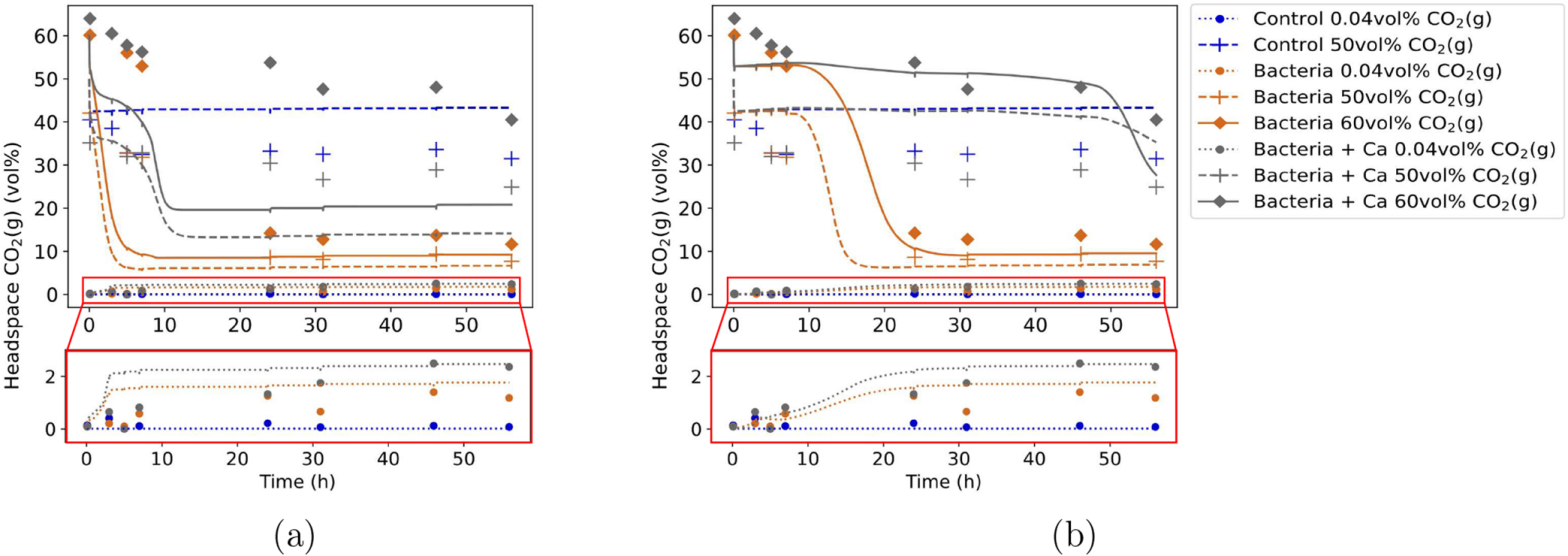
(a) Comparison of CO_2_(g) concentrations from experimental results with simulation of the batch experiments in bottles, with the simulation in (b) obtained with a 250 times lower bacterial concentration, giving a better alignment of simulation results with experiments. Experimentally measured values are shown as markers, simulation results as lines.

The initial sharp drop in volumetric CO_2_(g) fraction during the first 5 min of the simulations with 50 and 60 vol % initial CO_2_(g) concentration is attributed to the dissolution of CO_2_(g) in water. This dissolution occurs so quickly that the time lapse between injecting media into the bottle reactors and withdrawing the gas sample is likely sufficient for the drop to occur, resulting in no visible decrease in the experimental values in Figure 4a. Since the first measured value of “bacteria 60 vol % CO_2_(g)” and “bacteria + Ca 60 vol % CO_2_(g)” show around 60 vol % CO_2_(g), we presumed that the initial headspace CO_2_(g) concentration of the 60 vol % CO_2_(g) series was rather 70 vol % CO_2_(g).

Ureolysis does not occur in the “control” series, as the uncatalysed rate of [Disp-formula eq1] is 3.2 × 10^–21^ s^–1^,^17^ which is negligible. Therefore, the stronger decrease in headspace volumetric CO_2_(g) fraction of “bacteria 50 vol % CO_2_(g)” and “bacteria + Ca 50 vol % CO_2_(g)” compared to “control 50 vol % CO_2_(g)” provides experimental evidence of CO_2_(g) trapping by ureolysis, as shown in Figure 4a. This decrease in volumetric CO_2_(g) fraction is unlikely due to an increase in headspace NH_3_(g), as the simulations show negligible amounts of NH_3_(g) (less than ∼0.2 mM), while the molar CO_2_(g) concentration remains within 1–25 mM for the simulations with 50 and 60 vol % initial CO_2_(g) concentrations (Figure S4).

A larger amount of CO_2_(g) is trapped for “bacteria 50 vol % CO_2_(g)” and “bacteria 60 vol % CO_2_(g)” than for “bacteria + Ca 50 vol % CO_2_(g)” and “bacteria + Ca 60 vol % CO_2_(g)”, as shown in Figure 4a. This might seem counterintuitive, as the presence of Ca^2+^ offers additional potential for mineral trapping, with the expectation of a more pronounced decrease in CO_2_(g) fraction. Therefore, we examine below whether the trapped CO_2_(g) in “bacteria + Ca 50 vol % CO_2_(g)” and “bacteria + Ca 60 vol % CO_2_(g)” forms CaCO_3_ minerals or remains as soluble HCO_3_^–^ and CO_3_^2–^.

When experiments are conducted in air only (0.04% CO_2_(g)), bacterial activity introduces CO_2_(g) into the gas phase—a phenomenon that is noticeable in both the experimental and simulated results (Figure 4a). Thus, at sufficiently low CO_2_(g) concentrations, instead of trapping CO_2_(g), MICP leads to a release of CO_2_(g), attributed to originate from the H_2_CO_3_ produced during ureolysis ([Disp-formula eq2]). This finding is in good agreement with the study by Comadran-Casas et al.^51^ To understand the conditions that prevent this effect and hence promote CO_2_(g) trapping during MICP, we quantify the trapped CO_2_(g) relative to headspace CO_2_(g) and urea concentrations further below.

### Mineral Trapping

To determine if headspace CO_2_(g) could be trapped in the form of CaCO_3_ by MICP over an unrestricted duration, we calculated the concentration of different species at equilibrium in the “bacteria + Ca” series, thereby varying the initial concentration of Ca^2+^ and the headspace CO_2_(g) fraction. The headspace CO_2_(g) concentration was held constant, mimicking the unlimited CO_2_(g) supply when continuously injecting CO_2_(g) into geological CCS storage sites. All buffers were excluded to discuss the effects of MICP only. The details for calculating the equilibrium can be found in the SI, section S1.1.5.

The resulting [CaCO_3_] at equilibrium is plotted in Figure 5a. Increasing the initial CO_2_(g) fraction has a minimal impact on the amount of precipitated [CaCO_3_], which does not exceed 0.33 M, the same as the initial urea concentration in the solution (Figure 5b). Assuming full hydrolysis of urea at equilibrium, 0.33 M urea introduces 0.33 M C atoms as H_2_CO_3_, according to the ureolysis reactions 1 and 2. Thus, net mineral trapping of additional C atoms from headspace CO_2_(g) could occur only if the precipitated [CaCO_3_] exceeds 0.33 M, which is not the case.

**Figure 5:**
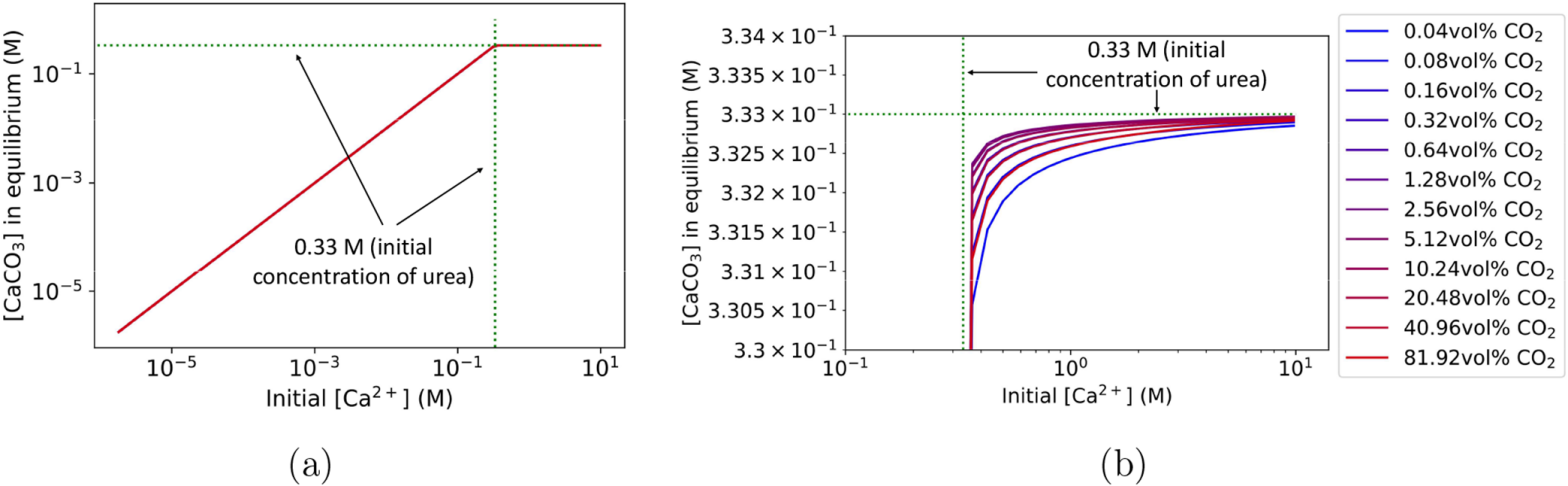
Concentration of (a) [CaCO_3_] at equilibrium for different initial [Ca^2+^] and CO_2_(g) fraction, with (b) being an enlarged version of (a) showing the CaCO_3_ concentration never exceeding 0.33 M. Buffers were not included in the liquid phase.

The limitation of [CaCO_3_] at equilibrium by the concentration of urea can be explained as follows. The hydrolysis of one urea molecule produces two NH_3_(aq) molecules, which can further react with water to produce two NH_4_^+^ molecules and two OH^–^ ions (R3). The extent of NH_3_(aq) reacting depends on the equilibrium of R3 and thus on [NH_4_^+^] and [OH^–^], i.e., pH. Furthermore, some NH_3_(aq) molecules also outgas (R4) (<1 mM for the cases in Figure 5, assuming equal volumes of headspace and liquid phases). Thus, with some NH_3_(aq) outgassing (R4) and not all NH_3_(aq) converting to NH_4_^+^ to produce OH^–^ (R3), the hydrolysis of one urea molecule produces two NH_3_(aq), leading to slightly less than two OH^–^ ions being generated per urea molecule hydrolyzed. In addition to producing two NH_3_(aq) molecules, the hydrolysis of one molecule of urea generates one molecule of H_2_CO_3_ ([Disp-formula eq1], 2). For mineral trapping to occur, H_2_CO_3_ must convert into CaCO_3_, thus requiring CO_3_^2–^ as an intermediate. This conversion needs two OH^–^ ions to counterbalance the two H^+^ ions introduced by H_2_CO_3_ (R5, R6). However, the production of less than two OH^–^ ions per urea molecule hydrolyzed does not leave any OH^–^ to support the hydrolysis of additional CO_2_(g) molecules into CaCO_3_.

This stoichiometric limitation is reflected in the pH response shown in Figure 6. The supply of slightly less than two OH^–^ ions per hydrolyzed urea molecule keeps the pH relatively constant for [Ca^2+^] below 0.33 M. However, once 0.33 M CaCO_3_ has precipitated for any initial [Ca^2+^] above 0.33 M (Figure 5a), the OH^–^ ions from ureolysis are depleted by the precipitation of 0.33 M CaCO_3_. Consequently, any further reaction between CO_3_^2–^ and Ca^2+^ causes a pH drop due to the lack of additional OH^–^ ions from ureolysis.

**Figure 6:**
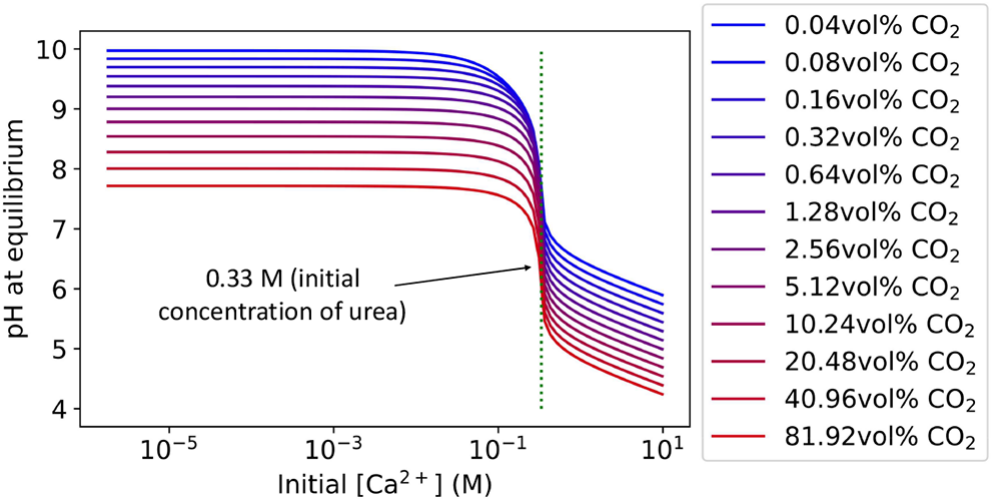
Corresponding pH to Figure 5 at equilibrium for varying initial [Ca^2+^] and CO_2_(g) fraction. Buffers were not included in the liquid phase.

This finding is in good agreement with that of Mitchell et al.,^28^ who reported no net precipitation of CO_2_(g) into CaCO_3_ by MICP in their modeled configuration. Therefore, we conclude that MICP alone does not lead to any mineral trapping of CO_2_(g) in the headspace. While the considered equilibrium calculations assume atmospheric pressure, the stoichiometric limitation is expected to persist at the higher pressures of geological CCS.

If the buffers from the media are included in the liquid phase, [CaCO_3_] can slightly exceed 0.33 M, reaching up to 0.345 M (Figure S5). This occurs because, as pH decreases after OH^–^ ions are depleted (Figure S6), the buffers from the media release additional OH^–^, providing OH^–^ for the precipitation of CaCO_3_ beyond the 0.33 M and thus leading to actual mineral trapping of CO_2_(g). However, using buffers, or more generally, enhancing alkalinity (i.e., the capacity of the liquid phase to neutralize acid^52^) to sequestrate CO_2_(g) is not a new idea, with ocean alkalinity enhancement (OAE) being one example. OAE generally aims to trap CO_2_(g) by converting CO_2_(g) into HCO_3_^–^ and CO_3_^2–^ (R8, R9, R5, R6) through increased alkalinity in the ocean, with various papers exploring OAE specifically.^52,53^ Thus, we will not further discuss buffer-based approaches but will focus exclusively on MICP.

#### Mineral Trapping in Sedimentary Reservoirs

Although we concluded that mineral trapping cannot be achieved with MICP alone, mineral trapping in actual sedimentary reservoirs is far more complex than the simple precipitation of CaCO_3_ ([Disp-formula eq3]), modeled here and in other MICP studies.^28,44,54^ Typically, mineral trapping in sedimentary reservoirs involves three steps:^55^

(i) first, CO_2_(g) is dissolved in water and forms HCO_3_^–^ and CO_3_^2–^ (R8, R9, R5, R6);
(ii) the resulting decrease in pH can lead to the dissolution of reactive sedimentary rock minerals, releasing metal ions (Mg^2+^, Fe^2+^, Al^2+^, and Ca^2+^);
(iii) the dissolution of the minerals and increase in metal ion concentration buffers and increases pH to basic conditions, favoring precipitation of carbonates as minerals.

Therefore, we propose a potential way to utilize MICP to accelerate the above-described steps of mineral trapping in sedimentary reservoirs. For step (i) of CO_2_(g) dissolution and subsequent carbonates formation, CO_2_(aq) hydrolysis (R9) has the lowest forward rate constant and is the limiting step among the reactions turning CO_2_(aq) into carbonates (R9, R5, R6).^49^ To investigate the effect of CA on the catalysis of R9, we simulated the CO_2_(g) dissolution and carbonate formation in media without urea and Ca^2+^ under a constant 50 vol % headspace CO_2_(g) fraction. The bacteria concentration was set to an OD_600_ of 5 (equivalent to 6.85 × 10^9^ CFU mL^–1^), i.e., an effective forward rate of 0.648 s^–1^ for R9.

The forward rate constant *k*_f,R8_ for CO_2_(g) dissolution in water (R8) depends on the mixing at the gas–liquid interface,^25^ which might vary depending on the location within a reservoir and the injection strategy. Therefore, we considered two scenarios:

(1) slow rate of R8, with *k*_f,R8_ = 2 × 10^–2^ s^–1^ as in Table 1,
(2) instantaneous CO_2_(g) dissolution, with *k*_f,R8_ = 1 × 10^10^ s^–1^.

The results for [HCO_3_^–^] and pH (Figure 7) show that equilibrium is reached within ∼3 min. When the forward rate constant *k*_f,R8_ for CO_2_(g) dissolution (R8) is slow, equilibrium is achieved at a similar rate, regardless of CA catalysis. In contrast, when (R8) is instantaneous, CA significantly reduces the time required to reach equilibrium. Thus, CA does not appear to be relevant for accelerating CO_2_(g) dissolution for a slow R8, as in scenarios with poor gas–liquid mixing, with further investigation required to determine the effectiveness of CA under such conditions in geological reservoirs. However, in systems where the gaseous and aqueous phases are only briefly in contact but strongly mixed, e.g., when a CO_2_(g) stream is bubbled into water for capturing the CO_2_(g) as carbonates, CA can enhance the dissolution rate of CO_2_(g) into the aqueous phase.

**Figure 7:**
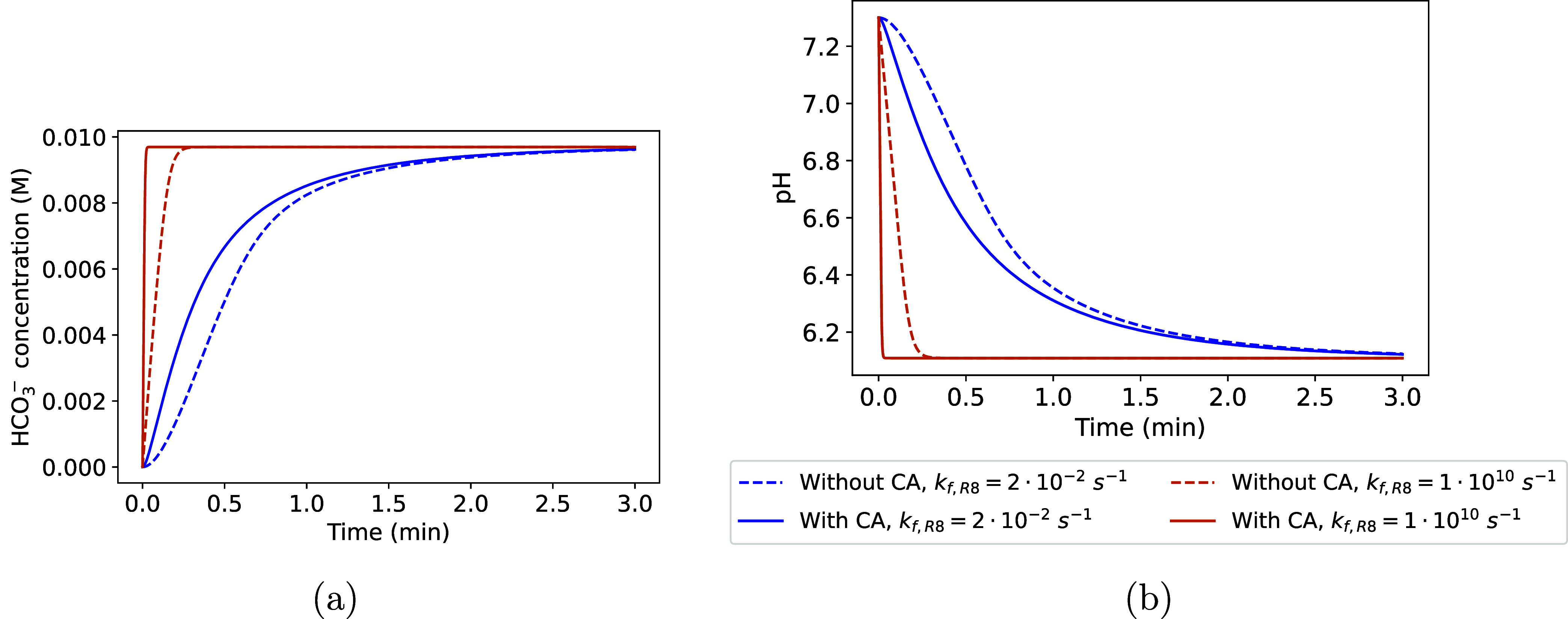
Simulation of (a) [HCO_3_^–^] and (b) pH during CO_2_(g) dissolution and carbonate formation with and without CA catalysis. The constant *k*_f,R8_ refers to the forward rate constant of R8.

Step (ii) involves the dissolution of sedimentary rock minerals, which releases metal ions (Ca^2+^, Al^3+^, Mg^2+^, *etc*.) and buffers the pH by consuming H^+^. The dissolution reactions are slow, occurring at rates of 10^–12^ to 10^–13^ mol m^–2^ s^–1^.^55^ Because of their complexity, we did not model this step. In the context of MICP, no previous research has targeted the rate of metal ion release, but we presume the rate is affected by MICP. We encourage future work to target the interaction of MICP with sedimentary rock minerals to understand if and under which conditions the interaction of MICP with sedimentary rock minerals could lead to CO_2_ mineral trapping.

For step (iii), we consider the effect of injecting bacteria and urea for MICP under the assumption of step (ii) having taken place, with metal ions having been dissolved into the aqueous phase and the pH buffered by the minerals. To simulate this, we implemented the model with a constant headspace CO_2_(g) fraction of 100 vol % to represent continued injection of CO_2_(g), 0.66 M Ca^2+^, and 0.66 M buffer with *pK*_*a*_ = 10 initialized at pH 10 to simulate the buffering capacity of the minerals. The high *pK*_*a*_ of 10 was chosen to minimize the interaction with pH during MICP in the simulation, while the initial pH of 10 ensures the buffer has the capacity to absorb H^+^. The equilibrium of the described system was calculated and set as the starting condition. At *t* = 0, we simulated the switching from injecting CO_2_(g) to bacteria and urea by suddenly increasing the urea concentration to 0.33 M (20 g L^–1^), and releasing the condition of constant headspace CO_2_(g) concentration and allowing the headspace CO_2_(g) concentration to adjust. The growth of bacteria and urease activity was modeled as for the experimental series “bacteria + Ca 60 vol % CO_2_(g)”, which is assumed to resemble the modeled case most closely among the 9 experimental series due to the high CO_2_(g) concentration and the presence of Ca^2+^.

At *t* < 0, the calculated starting condition is shown in Figure 8. The 100 vol % CO_2_(g), 0.66 M of Ca^2+^, and 0.66 M buffer have already caused some CaCO_3_ precipitation at equilibrium at *t* < 0 by converting CO_2_(g) to CaCO_3_ through the abiotic trapping pathway (R5–R9). The required OH^–^ ions for the trapping are supplied by the 0.66 M buffers. At *t* = 0, urea is injected and subsequently hydrolyzed within a few hours, leading to an increase in pH and a subsequent increase in CaCO_3_ precipitation and decrease in [Ca^2+^]. Although no additional mineral trapping of CO_2_(g) by MICP could be expected here, as discussed earlier, the introduction of urea and bacteria does significantly increase the pH from 5.2 to 8.1. As a result, [HCO_3_^–^] and [CO_3_^2–^] increase (Figure 8b), providing more ions for the precipitation of carbonate minerals with other metal ions in the liquid phase. The precise modeling of the buffering and dissolution of sedimentary rock minerals, as well as the effects of increased pH on carbonate mineral precipitation, requires further research. At this stage, MICP can be considered a potential method for controlling pH and consequently [HCO_3_^–^] and [CO_3_^2–^] in the mineral trapping process.

**Figure 8:**
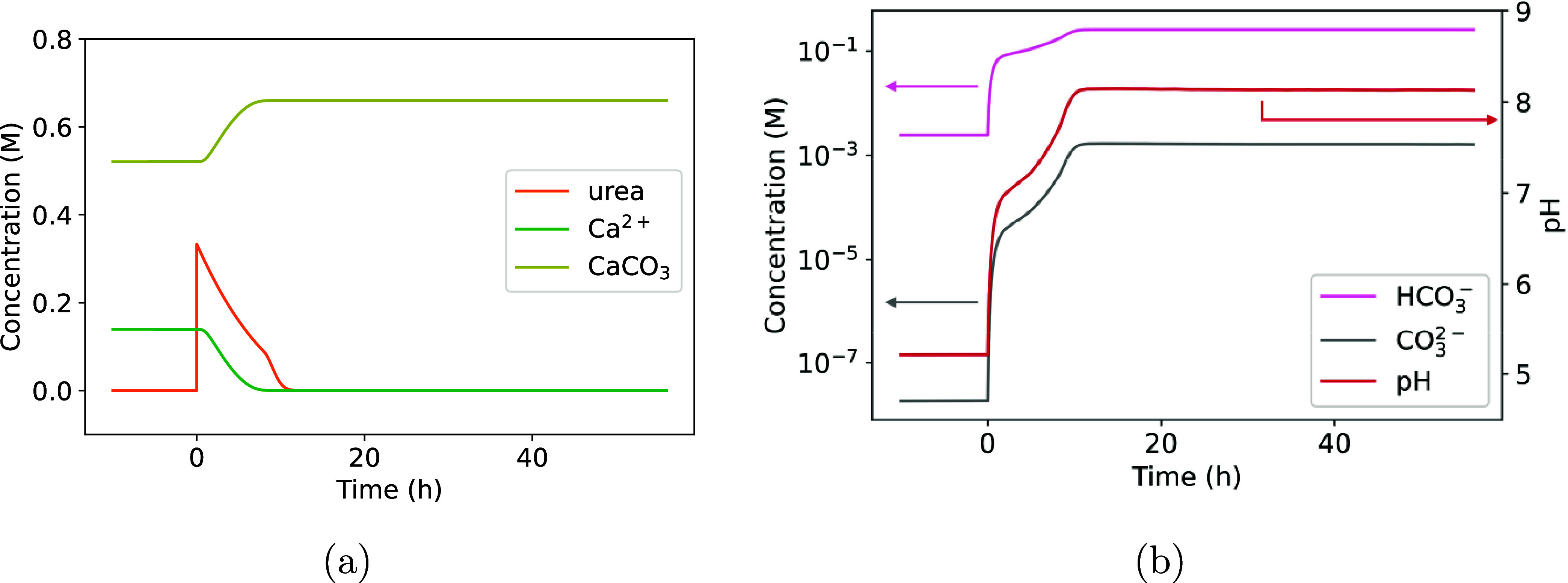
Simulation of the effect of MICP on (a) Ca^2+^, CaCO_3_, and urea concentration; (b) pH, [HCO_3_^–^], and [CO_3_^2–^] after mineral dissolution and pH buffering (step (iii)).

### Solubility Trapping

#### Maximum Trapped CO_2_(g)

Having explored the potential of MICP for mineral trapping in sedimentary reservoirs with reactive minerals, we also investigate the effects of ureolysis in reservoirs with low-reactivity minerals, where mineral trapping does not occur within millennia, or possibly at all.^55^ Such a scenario would be equivalent to setting [Ca^2+^] and [CaCO_3_] to zero in our model, resulting in solubility trapping as the only possible mechanism. As seen previously in the batch experiments (Figure 4), the headspace CO_2_(g) was observed to both decrease (in “bacteria 50 vol % CO_2_(g)” and “bacteria 60 vol % CO_2_(g)”) and increase (in “bacteria 0.04 vol % CO_2_(g)”). Moreover, the headspace of CO_2_(g) also decreases with ureolysis (Figure 4), suggesting that solubility trapping depends on the urea concentration or the amount of urea available in the liquid phase. Hence, we hypothesize that an optimum solubility trapping point exists, influenced by both the headspace CO_2_(g) fractions and urea concentration.

To determine the optimum for solubility trapping over an unrestricted duration, we calculated the concentration of different species in experiments containing bacteria without calcium at equilibrium, i.e., [Ca^2+^] and [CaCO_3_] set to zero. Moreover, the headspace CO_2_(g) concentration was held constant, mimicking the unlimited CO_2_(g) supply when continuously injecting CO_2_(g) into geological CCS storage sites.

Notably, the amount of trapped CO_2_(g) in the form of solubility trapping increases with higher urea concentration for headspace CO_2_(g) concentrations of at least 0.64 vol %, as shown in Figure 9a. Here, each line represents increasing headspace CO_2_(g) concentrations, with the respective maximum marked by a green point. These simulation results are in good agreement with experimental observations, where CO_2_(g) was trapped for “bacteria 50 vol % CO_2_(g)” and “bacteria 60 vol % CO_2_(g)”, while “bacteria 0.04 vol % CO_2_(g)” showed no CO_2_(g) trapping (Figure 4a).

**Figure 9:**
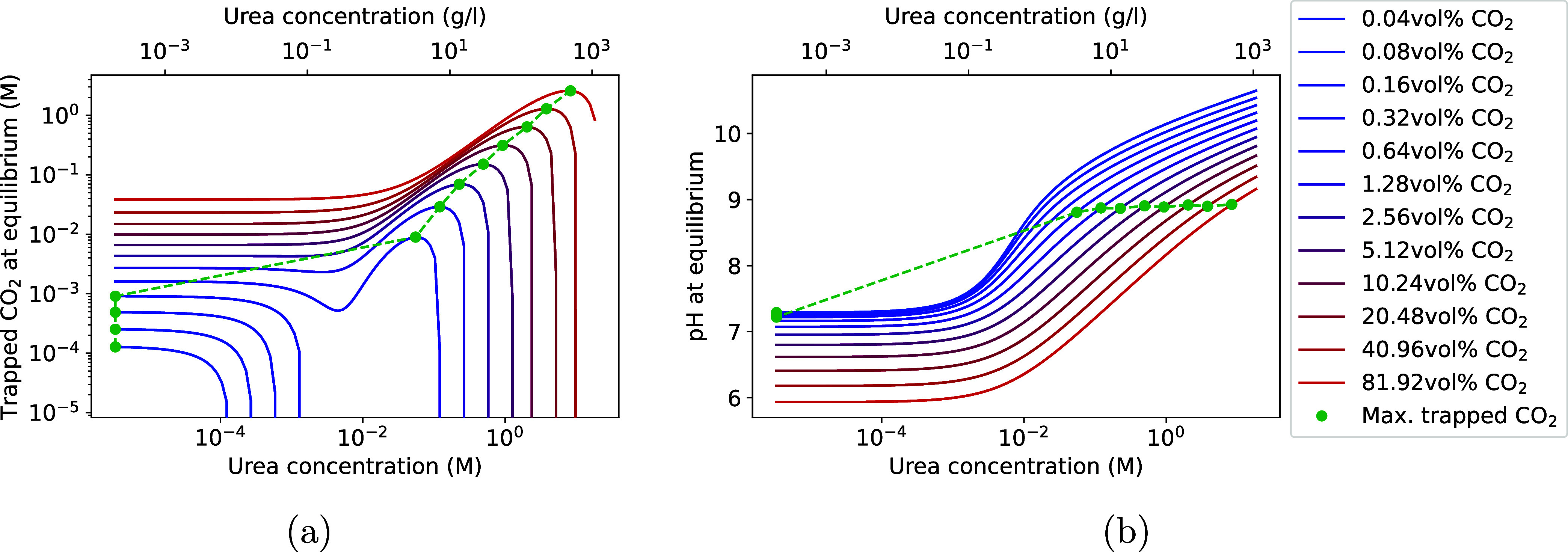
Simulation of (a) amount of trapped CO_2_(g) and (b) pH at equilibrium depending on urea and the CO_2_(g) headspace concentration. The green points indicate the maximum of the CO_2_(g) trapping for a given headspace CO_2_(g) concentration. A discontinuity in the green points is visible, as the maximum amount of trapped CO_2_(g) for a higher urea concentration than the minimum occurs only at CO_2_(g) fractions of at least 0.64 vol %.

For a given CO_2_(g) concentration, the increase in the amount of trapped CO_2_(g) with an increasing urea concentration can be explained by the elevated pH caused by higher urea concentrations (Figure 9b). The increase in pH leads to the production of HCO_3_^–^ and CO_3_^2–^ (R5 and R6), thereby enhancing solubility trapping. However, with a further increase in urea concentration and pH, ureolysis releases CO_2_(g) instead of trapping it, as shown in Figure 9a. This is because the hydrolysis of one urea molecule yields two NH_3_(aq) ions and one H_2_CO_3_ molecule ([Disp-formula eq1], 2). With NH_3_(aq) having an acidity constant of ∼9.25,^18^ two NH_3_(aq) molecules, on average, yield more than one OH^–^ ion by converting to NH_4_^+^ (R3) at pH < 9.25 (i.e., $\frac{\left[{\mathrm{NH}}_{4}^{+}\right]}{\left[{\mathrm{NH}}_{3}\left(\mathrm{aq}\right)\right]}>1$ ), while they yield less than one OH^–^ ion at pH > 9.25 (i.e., $\frac{\left[{\mathrm{NH}}_{4}^{+}\right]}{\left[{\mathrm{NH}}_{3}\left(\mathrm{aq}\right)\right]}<1$ ). For H_2_CO_3_ from ureolysis to convert into HCO_3_^–^ (R5), one OH^–^ ion is required to balance the release of one H^+^ ion, which is not the case for pH > 9.25. Therefore, not all H_2_CO_3_ produced from ureolysis can be converted to HCO_3_^–^, and the remaining H_2_CO_3_ must convert to CO_2_(aq) and eventually outgas as CO_2_(g). Thus, for pH > 9.25, increased ureolysis is expected to decrease the amount of trapped CO_2_(g), consistent with the observation of maximum trapped CO_2_(g) occurring around pH 8.9 (Figure 9b). Moreover, the fact that the maximum of CO_2_(g) trapping occurs at around pH 8.9 rather than pH 9.25 is attributed to some HCO_3_^–^ being further converted to CO_3_^2–^ at a higher pH (R6), releasing a H^+^ ion, which needs an additional OH^–^ ion for equilibration.

For headspace CO_2_(g) fractions below 0.64 vol %, trapped CO_2_(g) decreases at very low urea concentrations, where no increase in pH is observed (*cf*. Figure 9a,b). With no increase in pH, [HCO_3_^–^] and [CO_3_^2–^] remain constant, leading to H_2_CO_3_ from ureolysis outgassing in the form of CO_2_(g). This constant pH is ascribed to the buffers (HQa, ..., and HQg). In the absence of buffers, pH no longer remains constant at low urea concentrations, and a local maximum in trapped CO_2_(g) is observed for all CO_2_(g) headspace concentrations, as shown in Figure 10. It is thus crucial to account for the buffering capacity of the media, particularly at low urea concentrations, to avoid overestimating the amount of trapped CO_2_(g).

**Figure 10:**
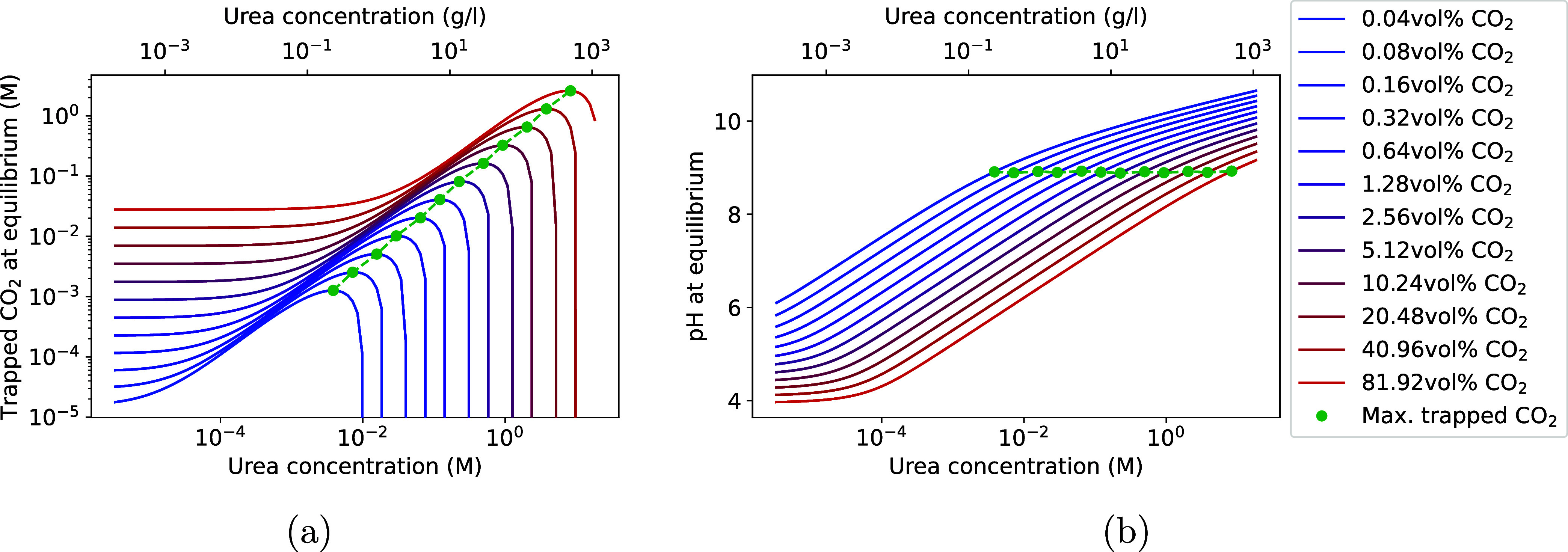
Simulation of (a) the amount of trapped CO_2_(g) and (b) pH at equilibrium depending on urea and the CO_2_(g) headspace concentration with buffers in the liquid phase being removed. The green points indicate the maximum of the CO_2_(g) trapping for a given headspace volumetric CO_2_(g) fraction.

#### Implication of Solubility Trapping in Reservoirs

To understand the potential and constraints of enhancing CO_2_(g) solubility trapping through ureolysis, we examine the maximal amount of trapped CO_2_(g) of the “bacteria” series (with and without buffer) across different urea concentrations at a given headspace CO_2_(g) concentration (Figure 11). This corresponds to maximal trapped CO_2_(g) indicated by the green points in Figure 9 (with buffer) and Figure 10 (without buffer). For comparison, we also show the maximal trapped CO_2_(g) in the “bacteria + Ca” series simulations across different urea concentrations at a given headspace CO_2_(g) concentration, i.e., adding 20 g L^–1^ CaCl_2_ to the “bacteria” series simulations. Furthermore, we calculated the trapped CO_2_(g) for the case of no ureolytic activity (as with the control experiments and termed “control” here) by setting the urea concentration to zero.

**Figure 11:**
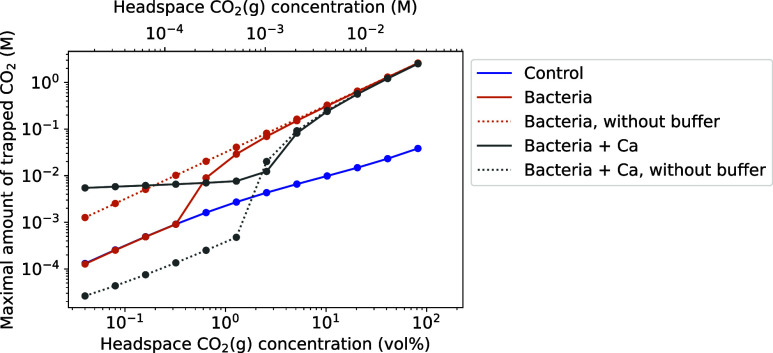
Maximal amount of trapped CO_2_(g) at equilibrium as determined in Figure 9, as well as calculated analogously for the “control” and “bacteria + Ca” experimental series. Lines are only a guide to the eye, whereas the points are actual simulated results.

For CO_2_(g) concentrations above 10 vol %, the maximum amount of trapped CO_2_(g) is highest for the “bacteria” series without buffer, with only a slight decrease when buffers are added (Figure 11). However, at atmospheric CO_2_(g) concentration (0.04 vol %), the “bacteria + Ca” series with buffers traps the largest amount of CO_2_(g) in comparison to the other series, attributed to the presence of buffers. Indeed, without buffers, “bacteria + Ca” traps the least CO_2_(g) at 0.04 vol %. In the “bacteria + Ca” series, the presence of Ca^2+^ decreases pH by consuming CO_3_^2–^ for CaCO_3_ precipitation ([Disp-formula eq3]), which releases H^+^ ions via R5 and R6 to balance the consumed CO_3_^2–^. This decrease in the pH reduces the capacity for HCO_3_^–^ and CO_3_^2–^ formation. However, the stronger decrease in pH of the “bacteria + Ca” series at 0.04 vol % CO_2_(g) compared to the “bacteria” series (compare Figures 9b and S7b, with Figure S7 showing the same simulation as Figure 9 but with 20 g L^–1^ CaCl_2_ in the liquid phase) enables a stronger release of OH^–^ by the buffers, which is suspected to lead, together with the CaCO_3_ precipitation, to an overall higher trapping of CO_2_(g). Therefore, the presence of buffers reduces the solubility trapping by ureolysis (Figure 11) by attenuating the necessary pH increase. However, for low urea concentrations and Ca^2+^ present in the liquid phase, buffers provide OH^–^ during the decrease of pH caused by Ca^2+^ and thus increase the amount of trapped CO_2_(g).

While the buffers included in the simulations are based on amino acids, the contact of CO_2_ with minerals in geological reservoirs can lead to pH buffering by mineral dissolution or no interaction with the minerals at all, with no pH buffering occurring.^55^ However, the comparisons in this study of simulations with and without buffers included in the liquid phase highlight the importance of considering any pH buffering when investigating MICP for CO_2_ trapping.

To examine the practical implications of these results, we focus on the “bacteria” series. At 82 vol % CO_2_(g), the “bacteria” series exhibits the second-largest maximum trapped CO_2_(g) compared to the other series, and contains the required growth medium (with buffers) for *S. pasteurii*. In a closed system, such as an aquifer, with a constant 82 vol % headspace CO_2_(g) concentration (0.033 M), ureolysis could trap up to 2.6 M CO_2_(g) as soluble HCO_3_^–^ and CO_3_^2–^ ions (Figure 11). This is nearly 80-fold higher than storing the CO_2_(g) as a gas (0.033 M) at the same headspace CO_2_(g) concentration, pressure, volume, and temperature, and 93-fold higher than storing the CO_2_(g) as soluble HCO_3_^–^ and CO_3_^2–^ ions in pure water at the same headspace CO_2_(g) concentration without ureolysis (0.028 M). Solubility trapping also offers the advantage of storing CO_2_(g) in the less labile form of soluble HCO_3_^–^ and CO_3_^2–^. However, the urea concentration of 497 g L^–1^ required to achieve maximal solubility trapping at 82 vol % headspace CO_2_(g) is neither industrially nor physically feasible and is used here for illustrative purposes. Accurate modeling of such high urea concentrations would require considering the solubility limit of all species in water, and the assumption of a constant concentration of water at 55.5 M would no longer hold.

To investigate a more realistic scenario, we consider urea-rich, industrial wastewater (e.g., the water formed during industrial urea synthesis and operations), containing a urea concentration of ∼20 g L^–1^ and regarded as a cheap and abundant source of urea.^28,40,41^ The amount of trapped CO_2_(g) at equilibrium for the :bacteria” series containing 20 g L^–1^ urea and with [CO_2_(g)] held constant is shown in Figure 12. Considering that Ca^2+^ is the most abundant cation in many groundwaters, results are also presented for bacteria with and without Ca^2+^, both when [Ca^2+^] is held constant and not, so that the presence of Ca^2+^ in reservoirs with less or nonreactive mineral rocks is examined as well. The lower amount of trapped CO_2_(g) in the presence of Ca^2+^ indicates a decrease in the efficacy of solubility trapping by ureolysis, and is explained by the consumption of CO_3_^2–^ for the formation of CaCO_3_. To maintain the equilibrium of $\frac{[{\mathrm{CO}}_{3}^{2-}]\left[H^{+}\right]}{\left[{\mathrm{HCO}}_{3}^{-}\right]}$ (R6), H^+^ are produced, reducing the pH and the capacity of the liquid for HCO_3_^–^ and CO_3_^2–^. The CO_3_^2–^ precipitated as CaCO_3_ (i.e., mineral trapping) is not expected to increase the level of CO_2_(g) trapping, as discussed previously. For the case with the highest amount of trapped CO_2_(g) in Figure 12, i.e., the “bacteria” series at 100 vol % CO_2_(g), the 0.347 M CO_2_(g) trapped by ureolysis constitutes an 8.6-fold increase compared to storing CO_2_(g) as gas at the same conditions (0.0402 M) and a more than 170-fold increase compared to the CO_2_(g) concentration stored naturally in the ocean surface (∼0.002 M).^56^ Similarly, pressure relief could be achieved. If CO_2_(g) is stored at a partial pressure of 8.6 atom, applying ureolysis could enable the storage of the same amount of CO_2_(g) at 1 atom, as schematically shown in Figure 13.

**Figure 12:**
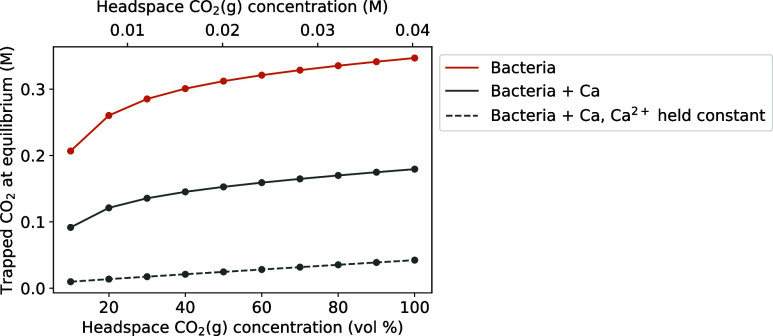
Amount of trapped CO_2_(g) at equilibrium (in form of HCO_3_^–^, CO_3_^2–^ and CaCO_3_) for 20 g L^–1^ urea depending on headspace CO_2_(g) concentration. Lines are only a guide for the eye, whereas the points are actual simulated results.

**Figure 13:**
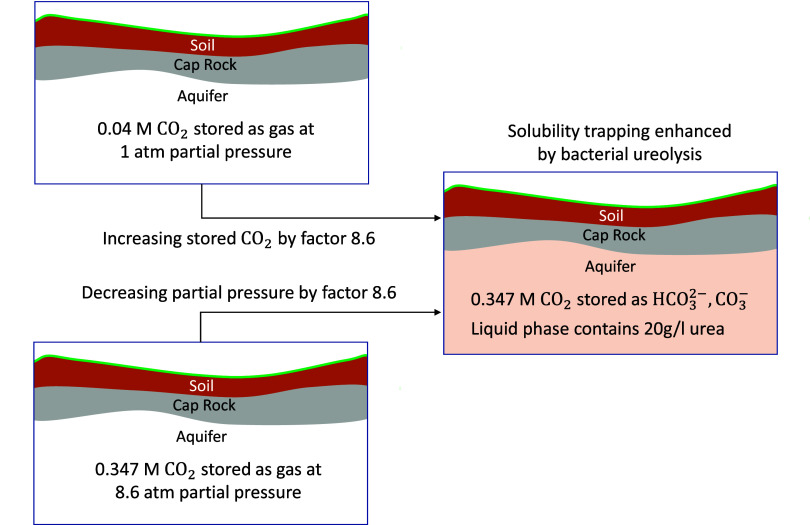
Possible effects of solubility trapping enhanced by bacterial ureolysis.

To determine the time required for trapping CO_2_(g) and equilibration, we simulated the batch experiments with the conditions of the “bacteria” series, maintaining a constant 100 vol % headspace CO_2_(g) concentration, shown in Figure 14. We conducted the simulation using the bacterial growth values from the “bacteria 60 vol % CO_2_(g)” (cf. Figure 3). The amount of trapped CO_2_(g) reaches an equilibrium concentration of 0.347 M within half a day, with the rate limited by ureolysis, i.e., pregrowing the bacteria could further increase the rate. Despite potential overestimation of the urease activity, this time scale, measured in days, is still in stark contrast to geological time scales, spanning years to millennia.

**Figure 14:**
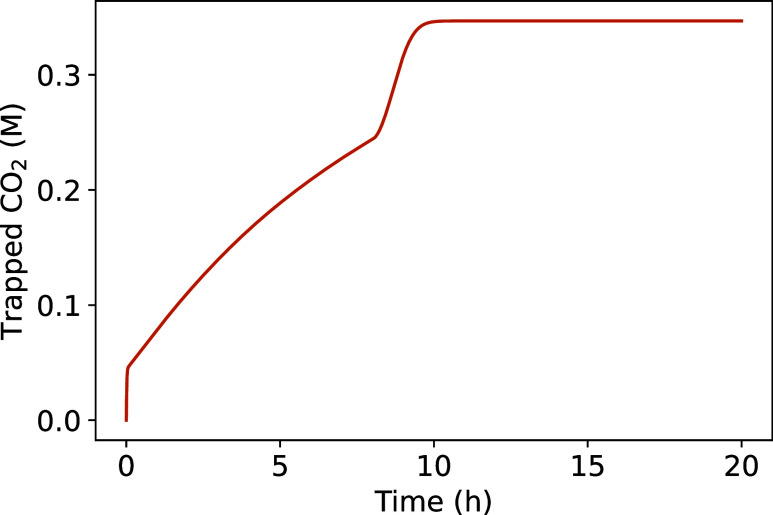
Simulation of “bacteria” series with a constant 100 vol % headspace CO_2_(g) concentration.

Therefore, if bacterial ureolysis is applied in geological formations, the storage capacity of solubility trapping could be increased in short time scales, opening up new opportunities for CO_2_(g) storage in less or nonreactive reservoirs. Additionally, the increased solubility trapping could serve as a critical intermediate step toward the safe, permanent storage of CO_2_(g) by facilitating subsequent mineral trapping through geochemical reactions,^32^ although further research is needed to understand the interaction between bacterial ureolysis and geochemical reactions. Furthermore, our results showed the influence of buffers on CO_2_ trapping, so future experiments should carefully consider the effects of buffers to avoid falsely attributing CO_2_ trapping from buffers to MICP, with both the buffering capacity from media and sedimentary rock minerals being relevant.

Although this study is limited to short-term experimental data, long-term MICP with the cyanobacteria *Synechococcus sp.* has been carried out successfully over one year by Dranseike et al.,^57^ demonstrating the general feasibility of MICP over the long term.

Worth reminding that the atmospheric pressure considered in this study is significantly lower than typical pressures in geological CCS sites (pressure above 73 atom and temperature above 31 °C).^28,32^ However, for *S. pasteurii*, Clarà Saracho et al.^30^ found that *S. pasteurii* exhibited only a slight decrease in growth and urease activity at 30 atom and 30 °C compared to 1 atom, suggesting its viability at geological CCS conditions. The equilibrium of the reactions ([Disp-formula eq1]–R19) and the physical properties of the species would need to be reconsidered at high pressures. Similarly, the obtained numerical values (time scales of reactions; absolute CO_2_(g) trapping values; urea concentration leading to maximal CO_2_(g) solubility trapping; increase in CO_2_(g) storage capacity using ureolysis compared to storing CO_2_ as a gas, or as HCO_3_^–^, CO_3_^2–^ in pure water or ocean surface) may change under geological CCS conditions. However, the key findings, as detailed below, are based on stoichiometry and remain valid independent of the temperature and pressure, unless explicitly stated for specific conditions.

### Key Findings

(i) Net mineral trapping of CO_2_(g), that is, the precipitation of C in excess of that derived from urea, did not occur within the range of simulated CO_2_(g) and initial Ca^2+^ concentrations. This is because, stoichiometrically, the precipitation of urea-derived C as CaCO_3_ consumes the two OH^–^ ions produced during ureolysis, making them unavailable for converting CO_2_(g) to CaCO_3_. At 0.04 vol % CO_2_(g) at 1 atom, MICP even causes CO_2_(g) to outgas.
(ii) The presence of Ca^2+^ lowers the efficacy of CO_2_(g) trapping by ureolysis, except at near atmospheric CO_2_(g) concentrations together with the presence of buffers.
(iii) MICP alone is unable to enhance CO_2_(g) mineral trapping. However, complex interactions with rock minerals in actual sedimentary reservoirs demand studying MICP in conjunction with sedimentary reservoir mechanisms, as stand-alone discussions on mineral trapping through MICP are insufficient.
(iv) The initial dissolution of CO_2_(g) during mineral trapping could be accelerated by the CA enzyme under strong gas–liquid phase mixing, while poor gas–liquid mixing reduces the accelerating effect of CA. Subsequently, the increase in pH, HCO_3_^–^, and CO_3_^2–^ induced by MICP might enhance mineral precipitation.
(v) In the absence of Ca^2+^, CO_2_(g) solubility trapping increases with urea concentration, until reaching a pH of ∼ 8.9. Further ureolysis at pH > 8.9 decreases the amount of solubility trapping, as one hydrolyzed molecule of urea produces insufficient OH^–^ ions for trapping the H_2_CO_3_ molecule from ureolysis. The limit for pH of 8.9 might change with temperature and pressure.
(vi) Buffers in the liquid medium decrease the amount of trapped CO_2_(g) by attenuating the pH increase from ureolysis. However, these buffers release OH^–^ if the pH decreases during CaCO_3_ precipitation at low headspace CO_2_(g) concentrations, thereby increasing CO_2_(g) trapping. Therefore, buffers should be carefully considered in experiments to avoid falsely attributing MICP instead of buffers as the cause of CO_2_ trapping.
(vii) Ureolysis opens up new opportunities for storing CO_2_ in less or nonreactive reservoirs via solubility trapping.

## Supplementary Material



## Notes

Methodology, additional experimental, and modeling results (PDF)

## References

[1] Masson-Delmotte, V. ; Zhai, P. ; Pörtner, H.-O. ; Roberts, D. ; Skea, J. ; Shukla, P. R. ; Pirani, A. ; Moufouma-Okia, W. ; Péan, C. ; Pidcock, R. Global warming of 1.5 C An IPCC Special Report on the impacts of global warming of 2018; Vol. 1, pp 43–50.

[2] Bachu S., Bonijoly D., Bradshaw J., Burruss R., Holloway S., Christensen N. P., Mathiassen O. M. (2007). CO2 storage capacity estimation:
Methodology and gaps. Int. J. Greenhouse Gas
Control.

[3] Lake, L. W. ; Lotfollahi, M. ; Bryant, S. L. Science of Carbon Storage in Deep Saline Formations; Elsevier, 2019; pp 15–31.

[4] Chiquier S., Patrizio P., Bui M., Sunny N., Mac Dowell N. (2022). A comparative
analysis of the efficiency, timing, and permanence of CO 2 removal
pathways. Energy Environ. Sci..

[5] Stavropoulou E., Laloui L. (2022). Evaluating CO2 breakthrough
in a shaly caprock material:
a multi-scale experimental approach. Sci. Rep..

[6] Kiyan T., Takade M., Namihira T., Hara M., Sasaki M., Goto M., Akiyama H. (2008). Polarity effect
in DC breakdown voltage
characteristics of pressurized carbon dioxide up to supercritical
conditions. IEEE Trans. Plasma Sci..

[7] Ndlovu P., Bulannga R., Mguni L. L. (2024). Progress
in carbon dioxide capture,
storage and monitoring in geological landform. Front. Energy Res..

[8] Chen Z., Zhou F., Rahman S. S. (2014). Effect
of cap rock thickness and
permeability on geological storage of CO2: laboratory test and numerical
simulation. Energy Explor. Exploit..

[9] Kelemen P., Benson S. M., Pilorgé H., Psarras P., Wilcox J. (2019). An overview
of the status and challenges of CO2 storage in minerals and geological
formations. Front. Climate.

[10] Feth, J. H. F. Preliminary map of the conterminous United States showing depth to and quality of shallowest ground water containing more than 1,000 parts per million dissolved solids. 1965.

[11] Blondes, M. S. ; Merrill, M. D. ; Anderson, S. T. ; DeVera, C. A. Carbon dioxide mineralization feasibility in the United States. 2019.

[12] Addassi M., Omar A., Hoteit H., Afifi A. M., Arkadakskiy S., Ahmed Z. T., Kunnummal N., Gislason S. R., Oelkers E. H. (2022). Assessing
the potential of solubility trapping in unconfined aquifers for subsurface
carbon storage. Sci. Rep..

[13] Ennis-King, J. ; Paterson, L. Greenhouse Gas Control Technologies - 6th International Conference; Gale, J. ; Kaya, Y. , Eds.; Pergamon: Oxford, 2003; pp 507–510.

[14] Paul V. G., Wronkiewicz D. J., Mormile M. R. (2017). Impact of elevated CO2 concentrations
on carbonate mineral precipitation ability of sulfate-reducing bacteria
and implications for CO2 sequestration. Appl.
Geochem..

[15] Rochelle C. A., Czernichowski-Lauriol I., Milodowski A. E. (2004). The impact
of chemical reactions on CO2 storage in geological formations: a brief
review. Geol. Soc. London Spec. Publ.

[16] Zeebe, R. E. ; Wolf-Gladrow, D. CO2 in Seawater: Equilibrium, Kinetics, Isotopes; Gulf Professional Publishing, 2001.

[17] Yao M., Tu W., Chen X., Zhan C.-G. (2013). Reaction pathways and free energy
profiles for spontaneous hydrolysis of urea and tetramethylurea: unexpected
substituent effects. Org. Biomol. Chem..

[18] Ammonia – Dissociation Constants https://pubchem.ncbi.nlm.nih.gov/compound/Ammonia#section=Dissociation-Constants&fullscreen=true (accessed May 07, 2022).

[19] Sander R. (2015). Compilation
of Henry’s law constants (version 4.0) for water as solvent. Atmos. Chem. Phys..

[20] Gibbons B. H., Edsall J. T. (1963). Rate of hydration of carbon dioxide
and dehydration
of carbonic acid at 25. J. Biol. Chem..

[21] Mitchell M. J., Jensen O. E., Cliffe K. A., Maroto-Valer M. M. (2010). A model
of carbon dioxide dissolution and mineral carbonation kinetics. Proc. R. Soc. A.

[22] Sezer, N. Production of precipitated calcium carbonate from marble wastes. M.Sc. Thesis, Middle East Technical University 2013.

[23] Maier U., Losen M., Büchs J. (2004). Advances in
understanding and modeling
the gas–liquid mass transfer in shake flasks. Biochem. Eng. J..

[24] Stillinger F. H. (1978). Proton
transfer reactions and kinetics in water. Theor.
Chem.: Adv. Perspect..

[25] Stumm, W. ; Morgan, J. J. Aquatic Chemistry: Chemical Equilibria and Rates in Natural Waters, 3rd ed.; John Wiley & Sons, 1996.

[26] Lauchnor E.
G., Topp D. M., Parker A. E., Gerlach R. (2015). Whole cell kinetics
of ureolysis by *Sporosarcina pasteurii*. J. Appl. Microbiol..

[27] Konstantinou C., Wang Y., Biscontin G., Soga K. (2021). The role of bacterial
urease activity on the uniformity of carbonate precipitation profiles
of bio-treated coarse sand specimens. Sci. Rep..

[28] Mitchell A. C., Dideriksen K., Spangler L. H., Cunningham A. B., Gerlach R. (2010). Microbially enhanced
carbon capture and storage by
mineral-trapping and solubility-trapping. Environ.
Sci. Technol..

[29] Pacheco V.
L., Bragagnolo L., Reginatto C., Thomé A. (2022). Microbially
Induced Calcite Precipitation (MICP): Review from an Engineering Perspective. Geotech. Geol. Eng..

[30] Clarà
Saracho A., Haigh S. K., Hata T., Soga K., Farsang S., Redfern S. A., Marek E. (2020). Characterisation of
CaCO3 phases during strain-specific ureolytic precipitation. Sci. Rep..

[31] Pearce J. K., La Croix A. D., Brink F. J., Hayes P. J., Underschultz J. R. (2021). CO2 mineral
trapping comparison in different regions: predicted geochemical reactivity
of the Precipice Sandstone reservoir and overlying Evergreen Formation. Pet. Geosci..

[32] Punnam P. R., Krishnamurthy B., Surasani V. K. (2022). Influence of Caprock Morphology on
Solubility Trapping during CO2 Geological Sequestration. Geofluids.

[33] Bozbeyoğlu N.
N., Candoğan T. Ş., Arslan Ş., Kabalay B., Bozkaya Ö., Akyol E., Doğan N. M. (2020). Calcium
carbonate precipitation by urease and carbonic anhydrase positive
bacteria. Pamukkale Üniversitesi Muhendislik
Bilimleri Derg..

[34] Heidarnezhad S., Wang S., Rahbar N. (2025). Carbonic Anhydrase
as a Sustainable
Corrosion Inhibitor for Concrete. ACS Appl.
Eng. Mater..

[35] Roberts B. P., Miller B. R., Roitberg A. E., Merz K. M. (2012). Wide-open flaps are key to urease activity. J. Am. Chem. Soc..

[36] Dhami N. K., Reddy M. S., Mukherjee A. (2014). Synergistic role of bacterial urease
and carbonic anhydrase in carbonate mineralization. Appl. Biochem. Biotechnol..

[37] Zheng T., Qian C. (2020). Influencing factors
and formation mechanism of CaCO3 precipitation
induced by microbial carbonic anhydrase. Process
Biochem..

[38] DSMZ Medium 220 https://www.dsmz.de/microorganisms/medium/pdf/DSMZ_Medium220.pdf (accessed May 14, 2022).

[39] Lapierre F. M., Schmid J., Ederer B., Ihling N., Büchs J., Huber R. (2020). Revealing nutritional requirements of MICP-relevant *Sporosarcina pasteurii* DSM33 for growth improvement
in chemically defined and complex media. Sci.
Rep..

[40] Rahimpour M., Mottaghi H., Barmaki M. (2010). Enhancement
of urea, ammonia and
carbon dioxide removal from industrial wastewater using a cascade
of hydrolyser–desorber loops. Chem. Eng.
J..

[41] Rittstieg K., Robra K.-H., Somitsch W. (2001). Aerobic treatment of a concentrated
urea wastewater with simultaneous stripping of ammonia. Appl. Microbiol. Biotechnol..

[42] Ghasemi P., Montoya B. M. (2022). Field implementation
of microbially induced calcium
carbonate precipitation for surface erosion reduction of a coastal
plain sandy slope. J. Geotech. Geoenviron. Eng..

[43] Gulay A., Fournier G., Smets B. F., Girguis P. R. (2023). Proterozoic acquisition
of archaeal genes for extracellular electron transfer: a metabolic
adaptation of aerobic ammonia-oxidizing bacteria to oxygen limitation. Mol. Biol. Evol..

[44] Clarà
Saracho A., Marek E. J. (2024). Uncovering the Dynamics of Urease
and Carbonic Anhydrase Genes in Ureolysis, Carbon Dioxide Hydration,
and Calcium Carbonate Precipitation. Environ.
Sci. Technol..

[45] Thomas K. C., Hynes S., Ingledew W. (2002). Influence of medium buffering capacity
on inhibition of Saccharomyces cerevisiae growth by acetic and lactic
acids. Appl. Environ. Microbiol..

[46] Tryptone https://grisp.pt/wp-content/uploads/2020/04/gcm23-tryptone.pdf (accessed Aug 06, 2022).

[47] Soy Peptone https://www.organotechnie.com/wp-content/uploads/2016/08/19649.pdf (accessed Aug 06, 2022).

[48] Chen, R. ; Kavala, A. M. ; Miller, E. ; Clarà Saracho, A. ; Marek, E. J. Measuring and Modelling Kinetics of CO2 hydration catalysed by Carbonic Anhydrase in Buffered Systems, ICBBG 2025 Proceedings, 2025.

[49] Mirjafari P., Asghari K., Mahinpey N. (2007). Investigating the application
of
enzyme carbonic anhydrase for CO2 sequestration purposes. Ind. Eng. Chem. Res..

[50] Myers J. A., Curtis B. S., Curtis W. R. (2013). Improving accuracy of cell and chromophore
concentration measurements using optical density. BMC Biophys..

[51] Comadran-Casas C., Brüggemann N., Jorat M. E. (2024). Greenhouse gas fluxes of microbial-induced
calcite precipitation at varying urea-to-calcium concentrations. Eur. J. Soil Sci..

[52] Renforth P., Henderson G. (2017). Assessing
ocean alkalinity for carbon sequestration. Rev.
Geophys..

[53] Hartmann, J. ; Suitner, N. ; Lim, C. ; Schneider, J. ; Marín-Samper, L. ; Arístegui, J. ; Renforth, P. ; Taucher, J. ; Riebesell, U. Stability of alkalinity in ocean alkalinity enhancement (OAE) approaches–consequences for durability of CO_2_ storage Biogeosciences Discussions 2022; Vol. 2022, pp 1–29.

[54] Phillips A. J., Gerlach R., Lauchnor E., Mitchell A. C., Cunningham A. B., Spangler L. (2013). Engineered applications of ureolytic biomineralization:
a review. Biofouling.

[55] Khandoozi S., Hazlett R., Fustic M. (2023). A critical
review of CO2 mineral
trapping in sedimentary reservoirs–from theory to application:
Pertinent parameters, acceleration methods and evaluation workflow. Earth-Sci. Rev..

[56] Feely R. A., Sabine C. L., Takahashi T., Wanninkhof R. (2001). Uptake and storage of carbon dioxide in the
ocean: The global cõ
2 survey. Oceanography.

[57] Dranseike D., Cui Y., Ling A. S., Donat F., Bernhard S., Bernero M., Areeckal A., Lazic M., Qin X.-H., Oakey J. S. (2025). Dual carbon sequestration with photosynthetic living materials. Nat. Commun..

