# Supplementary material for: Modeling the Effect of Microbially Induced Calcium Carbonate Precipitation (MICP) on CO2 Trapping
Source: Environ Sci Technol. 2025 Oct 2;59(40):21486–500. doi: 10.1021/acs.est.5c08890 (PMC12529959; doi:10.1021/acs.est.5c08890)
Supplement: Supplementary file 1 [file es5c08890_si_001.pdf]

Supporting Information

Modelling the effect of microbially induced calcium carbonate precipitation (MICP) on CO_2 trapping

Raymond Chen,*[a] Ahmet Mert Kavala,[b] Alexandra Clarà Saracho[b] and Ewa J. Marek*[a]

[a] *University of Cambridge, Department of Chemical Engineering and Biotechnology, West Cambridge Site, Philippa Fawcett Drive, Cambridge, CB3 0AS, United Kingdom.*

[b] *The University of Texas at Austin, Department of Civil, Architectural and Environmental Engineering, 301 E. Dean Keeton St., Austin, TX 78712, United States.*

* *Corresponding authors. E-mail: rc854@cam.ac.uk, ejm94@cam.ac.uk*

Outline

The supporting information file contains 11 pages, which include 7 Figures and 1 Table.

Contents

S1 Supplementary Methods **S3**

S1.1 Modelling of substance concentrations during MICP S3

S2 Supplementary Figures **S7**

List of Figures

S1 Comparison of the experimental titration curve of DSMZ 220 media and the best fit (a) with and (b) without buffer 7, respectively, for determining the buffer concentrations. Adding buffer 7 increases the accuracy of the fit visibly, but likely not crucially. The concentration of buffer 1-6 is not the same for Fig. (a) and (b), as Fig. (b) shows the best obtained fit for using only buffer 1-6. S7

S2 Experimentally determined first-order rate constant $k_{f,CA}$ of reaction R9. . . S7

S3 Comparison of (a) the original plot of species concentration in a carbonate system from Mitchell *et al.* [1] with (b) the plot recreated with our model. Ca_0^{2+} refers to the initial concentration of Ca^{2+}. S8

S4 Simulation of the (molar) (a) [CO_2(g)] and (b) [NH_3(g)] concentration of the batch experiments in bottles. S8

S5 Concentration of (a) [$CaCO_3$] at equilibrium for different initial [Ca^{2+}] and CO_2(g) fraction, with (b) being an enlarged version of (a) – showing the $CaCO_3$ concentration slightly exceeding 0.33 M. Media buffers were included in the liquid phase. S9

S6 Corresponding pH to Fig. S5 in equilibrium for varied initial [Ca^{2+}] and CO_2(g) fraction. S9

S7 Simulation of (a) amount of trapped CO_2(g) and (b) pH at equilibrium depending on urea and CO_2(g) headspace concentration with 20 g/L $CaCl_2$ and buffers in the liquid phase. The green points indicate the maximum of CO_2(g) trapping for a given headspace volumetric CO_2(g) fraction. S10

List of Tables

S1 Buffer symbols, pK_a and total concentration. S7

S1 Supplementary Methods

S1.1 Modelling of substance concentrations during MICP

S1.1.1 Initial conditions of the model.

The initial pressure was fixed at atmospheric pressure, with the initial molar concentration of CO_2(g) (the only pressure-dependent parameter) calculated *via* the ideal gas equation with a pressure of 1013.25 hPa and temperature of 30 °C, matching the temperature in the shaking incubator. Air was considered inert and, thus, was not modelled explicitly. The liquid phase was assumed to have a constant density of 1 kg L^{-1}, with water as a dominating component at a constant concentration of 55.5 M, included in the rate and equilibrium constants of H_2O-involving reactions.

S1.1.2 Determining buffer concentration.

First, the pH of DSMZ 220 media containing 20 g L^{-1} urea was increased to pH 10 using 0.1 M NaOH solution, then, the mixture was titrated using a Metrohm 848 Titrino plus automatic titrator. A simulated titration curve of a titrand containing 6 buffers was fitted to the experimental titration curve. The fitted parameter was the total molar concentration of each buffer, representing the sum of its conjugate base and acid. The pK_a values of these buffers were selected to cover a wide pH range and were based on the pK_a values of amino acids present in DSMZ 220 media, assuming no other components contributed to the buffering capacity of the media. The selected pK_a values were 9.7, 8.3, 6.0, 4.2, 3.9, and 2.4, corresponding to aspartic acid, glutamic acid, histidine, cysteine, and leucine, respectively [2, 3, 4]. The molar concentration of each buffer was obtained by manually minimising the squared difference between the simulated and experimental titration curves. Because no set of concentrations based on the 6 amino acid pK_a values fit the titration curve to a satisfactory degree (Fig. S1b in the electronic supporting information (SI)), an additional seventh buffer with $pK_a = 7.1$ was added. This increased the accuracy of the fit presented in Fig. S1 while allowing to account for potential unidentified substances or interactions among the various components present in the media. Still, the source of buffer 7 remains unclear. The seven buffers, denoted Qa^-, Qb^-, ..., Qg^-, are listed with their conjugate bases in Table S1, and their corresponding dissociation reactions (R13-R19) are listed in Table 1.

The initialisation of the buffers involved a combination of mass conservation and equilibrium equations. For example, the buffer Qa^- was initialised by solving the following equations for the initial pH value:

$$[\mathrm{Qa}^-] + [\mathrm{HQa}] = c_{\mathrm{Qa}^-} \tag{S1}$$

$$\frac{[H^+][Qa^-]}{[HQa]} = 10^{-pK_{a,Qa^-}} \tag{S2}$$

where pK_{a,Qa^-} is the acid dissociation constant, and c_{Qa^-} is the total molar concentration of the buffer Qa^-.

S1.1.3 Reaction rates of CA-catalysed reactions.

The rate of the CA-catalysed (R9) can be described by the Michaelis-Menten kinetics [5], which approximates to first-order reaction kinetics at low substrate and product concentrations. To determine the first-order kinetics of CA, *S. pasteurii* (ATCC 11859) was cultivated in DSMZ 220 media with $20\,g\ L^{-1}$ urea. At the mid-exponential growth phase (OD_{600} of 0.45), the bacterial culture was centrifuged, and the resulting pellet was washed twice with 1X PBS buffer solution (Thermo Fisher Scientific, J67802.AP). Subsequently, the pellet was resuspended in PBS buffer to obtain two sample solutions with OD_{600} values of 0.5 and 1.6. A negative control of only PBS buffer solution was prepared as well. The sample solutions were introduced into a 96-well plate together with phenol-red indicator and a CO_2(aq) solution, prepared by bubbling CO_2(g) in DI water. The CO_2(aq) solution reacted according to R9 and R5, releasing H^+ and thus decreasing pH. The change in pH was accompanied by a colour change in the phenol red, which was monitored by a plate reader (Biotek Synergy H1), through which the pH was determined using a standard curve. A modelled pH response was subsequently fitted to the experimental pH measurements, yielding the first-order rate constant of the CA-catalysed R9. The buffering capacity of the 9.56 mM phosphates in the PBS buffer solution (KH_2PO_4, Na_2HPO_4) was considered in the modelled pH response, with the three pK_a values of phosphate being 2.148, 7.198, 12.35 [6]. Simulations fitted to experimental titration curves (Thermo Fisher Scientific, Orion Star T910) of the resuspended bacterial pellet identified an additional buffer with a pK_a of 6 and a concentration of 0.5 mM, likely originating from components in the pellet. This additional buffer was considered in the modelled pH response for determining the rate of the catalysed R9, but was excluded from simulations of the batch experiments in bottles.

The correlation between OD_{600} and the first-order rate constant of R9 is shown in Fig. S2 and was assumed to be linear, yielding the formula

$$k_{f,CA} = 0.065\,s^{-1} + 0.117\,s^{-1} \cdot [X_{OD}] \tag{S3}$$

with $[X_{OD}]$ being the cell concentration expressed in OD_{600}.

The intercept of $0.065\,s^{-1}$ accounts for the rate of the uncatalysed R9, aligning closely with reported literature values of 0.03-0.062 s^{-1} [1, 5, 7]. The above methods for determining the first-order rate constant of the CA-catalysed R9 are detailed by Chen *et al.* [8].

S1.1.4 Simulation of experiments in bottle reactors and of results from literature.

During the batch experiments in bottle reactors, the withdrawal of 5 mL gas sample and 2 mL liquid sample were modelled to decrease the mole per volume concentration of all species in the gas phase by a factor $\frac{V_g}{V_g+7\,\mathrm{mL}}$, with V_g being the volume of the gas phase. For the liquid phase, the withdrawal of 2 mL liquid sample did not change the mole per volume concentration of aqueous species.

We validated our model by comparing the modelled temporal changes in species concentrations within a carbonate system (CO_2(g), CO_2(aq), HCO_3^-, H_2CO_3, CO_3^{2-}, H^+, Ca^{2+}, $CaCO_3$, and water) with the results from Mitchell *et al.* [1]. We set rate and equilibrium constants, and initial values, to the same values used by Mitchell *et al.* [1], while the rate constants for reactions not modelled by Mitchell *et al.*[1] were set to zero and buffers were excluded. As shown in Fig. S3, our simulated results closely align with those of Mitchell *et al.*, demonstrating that our model reproduces results from literature accurately, and confirming the validity of the assumptions used to model the carbonate system without buffers. However, as discussed earlier, accounting for the buffering capacity of media is essential to depict the conditions of the MICP process correctly.

S1.1.5 Simulation of equilibrium.

To investigate mineral and solubility trapping under continuous CO_2(g) injection and supply in geological CCS storage sites, we determined the limits of mineral and solubility trapping by calculating the species concentration at thermodynamic equilibrium. Since the hydrolysis of urea is considered irreversible [9], urea and NH_2COOH were assumed to be fully hydrolysed at equilibrium, yielding NH_3(aq) and H_2CO_3. The concentrations of the remaining 19 species were determined by solving the equation system derived from the equilibrium of R3-R19, excluding R11 and R12, by equating the equilibrium constant K to the ratio of the species participating in the reaction (Eq. 2). Reactions R11 and R12 represent alternate pathways of R9, R5, and R6; thus, their equilibrium expressions are equivalent to those derived from R9, R5, and R6. In addition, four boundary conditions were imposed: constant CO_2(g) concentration for simulating the continuous CO_2(g) supply (Eq. S4), conservation of the number of nitrogen atoms (Eq. S5), conservation of the number of calcium atoms (Eq. S6), and conservation of charge (Eq. S7),

$$c_{\mathrm{CO_2}(g)} = [\mathrm{CO_2}(g)] \tag{S4}$$

$$2c_{\mathrm{urea}} = [\mathrm{NH_3}(g)] + [\mathrm{NH_3}(aq)] + [\mathrm{NH_4}^+] \tag{S5}$$

$$c_{\mathrm{Ca^{2+}}} = [\mathrm{Ca^{2+}}] + [\mathrm{CaCO_3}] \tag{S6}$$

$$2c_{\mathrm{Ca^{2+}}} + c_{\mathrm{H^+}} - c_{\mathrm{OH^-}} - c_{\mathrm{Qa^-}} - c_{\mathrm{Qb^-}} - c_{\mathrm{Qc^-}} - c_{\mathrm{Qd^-}} - c_{\mathrm{Qe^-}} - c_{\mathrm{Qf^-}} - c_{\mathrm{Qg^-}}$$

$$
\begin{aligned}
&= 2[Ca^{2+}] + [H^+] - [OH^-] - [HCO_3{}^-] - 2[CO_3{}^{2-}] + [NH_4{}^+] \\
&- [Qa^-] - [Qb^-] - [Qc^-] - [Qd^-] - [Qe^-] - [Qf^-] - [Qg^-],
\end{aligned} \tag{S7}
$$

where c is the initial concentration of the species in the index or a constant concentration in the case of $c_{CO_2(g)}$.

S2 Supplementary Figures

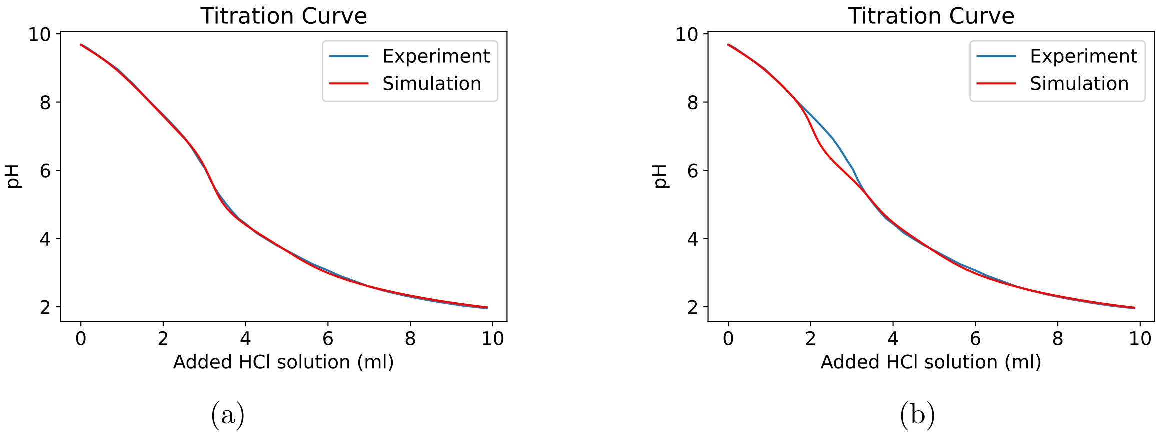

Figure S1: Comparison of the experimental titration curve of DSMZ 220 media and the best fit (a) with and (b) without buffer 7, respectively, for determining the buffer concentrations. Adding buffer 7 increases the accuracy of the fit visibly, but likely not crucially. The concentration of buffer 1-6 is not the same for Fig. (a) and (b), as Fig. (b) shows the best obtained fit for using only buffer 1-6.

Table S1: Buffer symbols, pK_a and total concentration.

	Symbol	pK_a	Total concentration (M)
Buffer 1	HQa, Qa^-	9.7	0.027
Buffer 2	HQb, Qb^-	8.3	0.011
Buffer 3	HQc, Qc^-	6.0	0.0035
Buffer 4	HQd, Qd^-	4.2	0.022
Buffer 5	HQe, Qe^-	3.9	0.003
Buffer 6	HQf, Qf^-	2.4	0.046
Buffer 7	HQg, Qg^-	7.1	0.0135

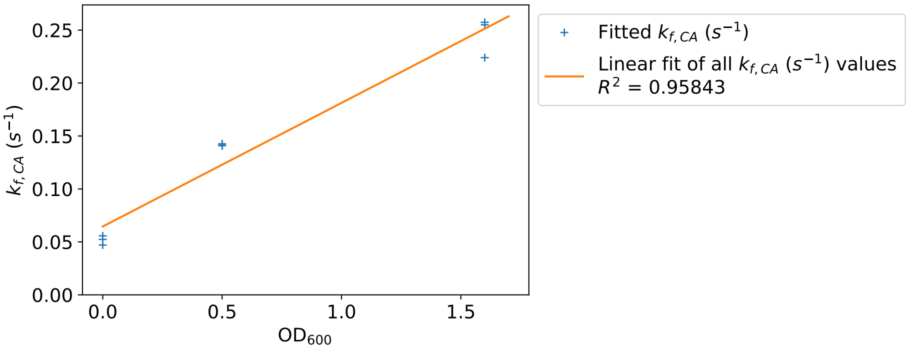

Figure S2: Experimentally determined first-order rate constant $k_{f,CA}$ of reaction R9.

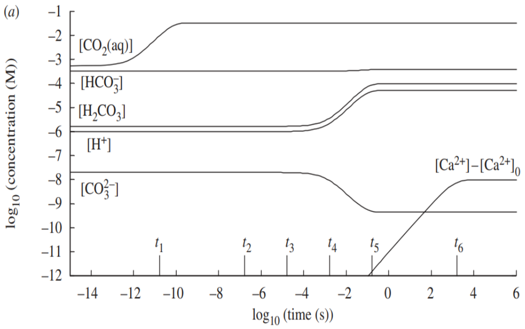

(a)

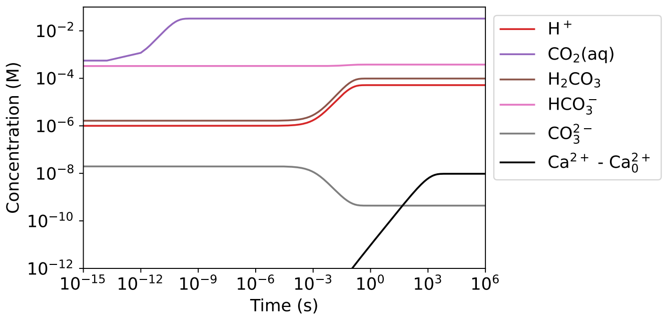

(b)

Figure S3: Comparison of (a) the original plot of species concentration in a carbonate system from Mitchell *et al.* [1] with (b) the plot recreated with our model. Ca_0^{2+} refers to the initial concentration of Ca^{2+}.

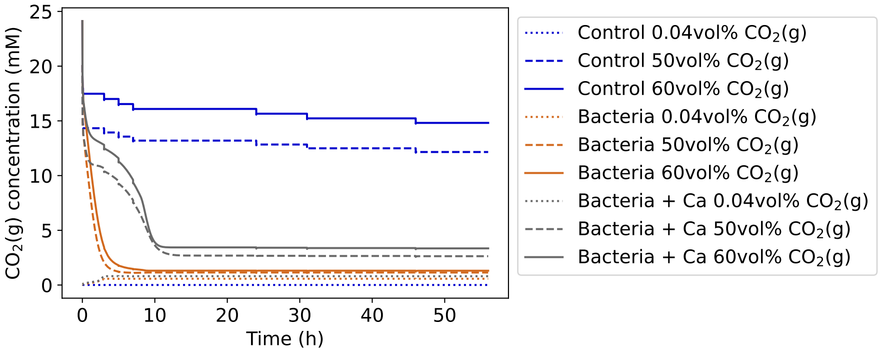

(a)

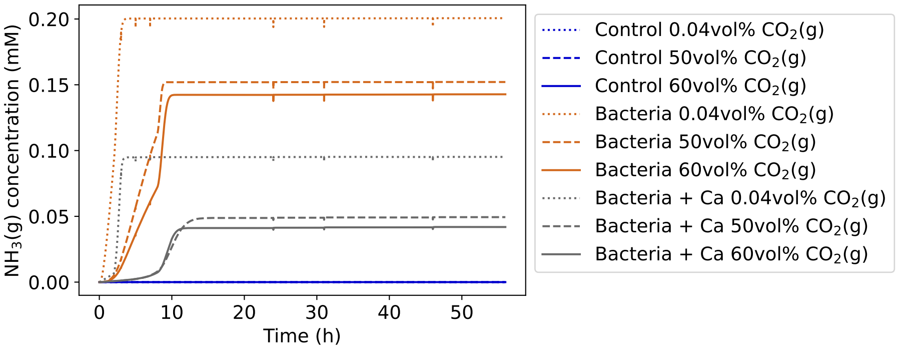

(b)

Figure S4: Simulation of the (molar) (a) $[CO_2(g)]$ and (b) $[NH_3(g)]$ concentration of the batch experiments in bottles.

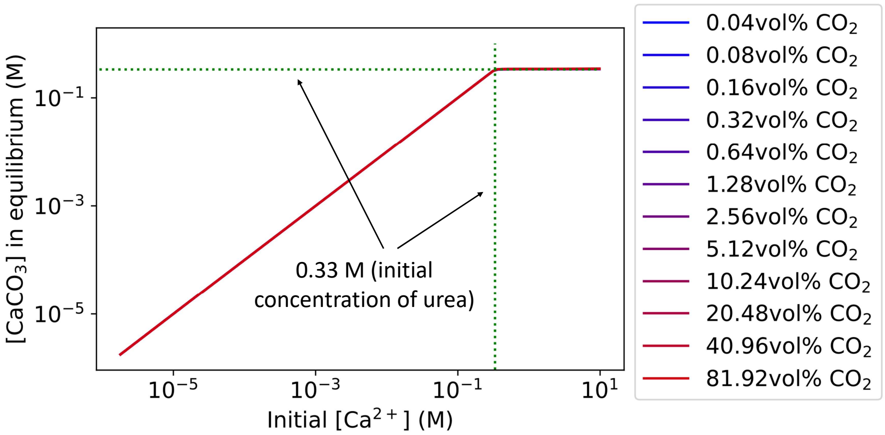

(a)

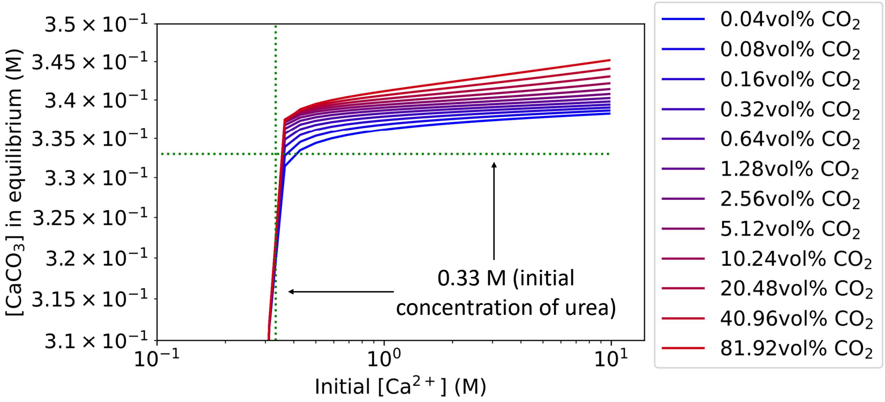

(b)

Figure S5: Concentration of (a) $[CaCO_3]$ at equilibrium for different initial $[Ca^{2+}]$ and CO_2(g) fraction, with (b) being an enlarged version of (a) – showing the $CaCO_3$ concentration slightly exceeding 0.33 M. Media buffers were included in the liquid phase.

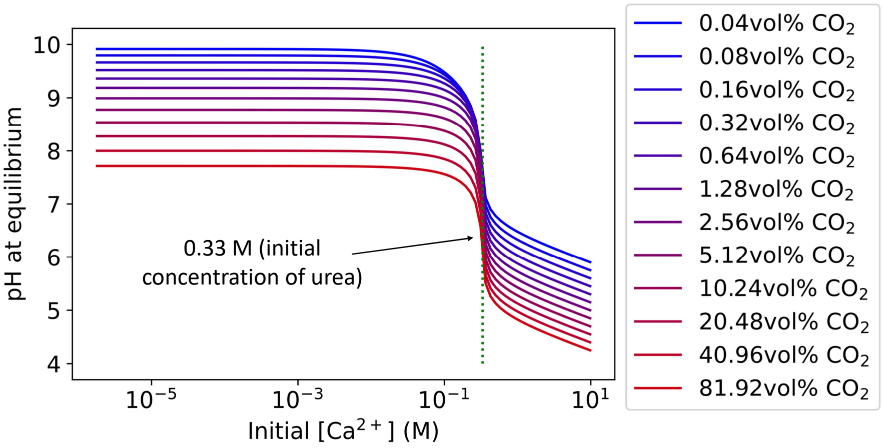

Figure S6: Corresponding pH to Fig. S5 in equilibrium for varied initial $[Ca^{2+}]$ and CO_2(g) fraction.

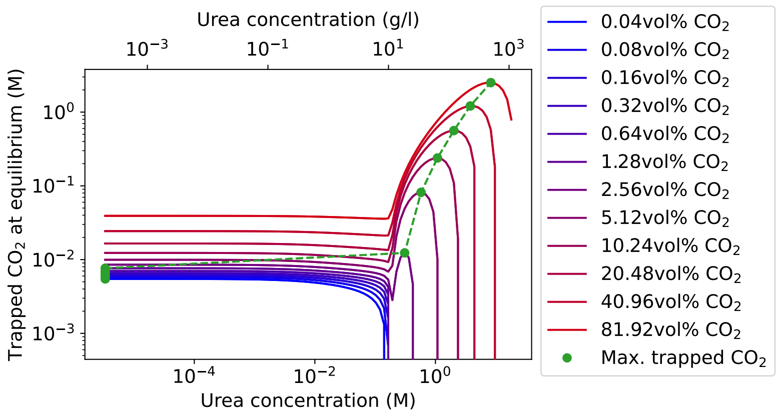

(a)

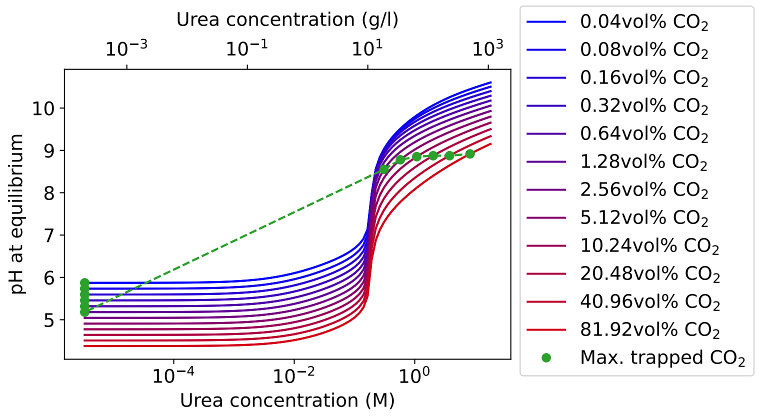

(b)

Figure S7: Simulation of (a) amount of trapped CO_2(g) and (b) pH at equilibrium depending on urea and CO_2(g) headspace concentration with 20 g/L $CaCl_2$ and buffers in the liquid phase. The green points indicate the maximum of CO_2(g) trapping for a given headspace volumetric CO_2(g) fraction.

References

[1] M. J. Mitchell, O. E. Jensen, K. A. Cliffe, and M. M. Maroto-Valer, "A model of carbon dioxide dissolution and mineral carbonation kinetics," *Proceedings of the Royal Society A: Mathematical, Physical and Engineering Sciences*, vol. 466, no. 2117, pp. 1265–1290, 2010.

[2] C. Mathews, K. Van Holde, and K. Ahern, *Biochemistry, Third Edition.* Addison-Wesley world student series, Benjamin Cummings, 2000.

[3] "Tryptone." `https://grisp.pt/wp-content/uploads/2020/04/gcm23-tryptone.pdf`. Accessed: 06-08-2022.

[4] "Soy peptone." `https://www.organotechnie.com/wp-content/uploads/2016/08/19649.pdf`. Accessed: 06-08-2022.

[5] P. Mirjafari, K. Asghari, and N. Mahinpey, "Investigating the application of enzyme carbonic anhydrase for co2 sequestration purposes," *Industrial & engineering chemistry research*, vol. 46, no. 3, pp. 921–926, 2007.

[6] R. N. Goldberg, N. Kishore, and R. M. Lennen, "Thermodynamic quantities for the ionization reactions of buffers," *Journal of physical and chemical reference data*, vol. 31, no. 2, pp. 231–370, 2002.

[7] W. Stumm and J. J. Morgan, *Aquatic chemistry: chemical equilibria and rates in natural waters, 3rd edition.* John Wiley & Sons, 1996.

[8] R. Chen, A. M. Kavala, E. Miller, A. Clarà Saracho, and E. J. Marek, "Measuring and modelling kinetics of co2 hydration catalysed by carbonic anhydrase in buffered systems," *ICBBG 2025 Proceedings*, 2025.

[9] C. Konstantinou, Y. Wang, G. Biscontin, and K. Soga, "The role of bacterial urease activity on the uniformity of carbonate precipitation profiles of bio-treated coarse sand specimens," *Scientific reports*, vol. 11, no. 1, pp. 1–17, 2021.
